# Modulating Local Coordination in Single‐Atom Catalysts: From Fundamental Concepts to Emerging Breakthroughs in Electrocatalysis

**DOI:** 10.1002/anie.3059499

**Published:** 2026-08-05

**Authors:** Ashwani Kumar, Harun Tüysüz

**Affiliations:** ^1^ Department of Heterogeneous Catalysis Max‐Planck‐Institut für Kohlenforschung Mülheim an der Ruhr Germany; ^2^ Centre for Nanotechnology Indian Institute of Technology Roorkee Roorkee Uttarakhand India; ^3^ Catalysis and Energy Materials IMDEA Materials Institute Getafe Madrid Spain

**Keywords:** catalytic mechanism, electrocatalysis, electronic descriptor, local microenvironment, single atom catalysts

## Abstract

Single‐atom catalysts (SACs) have attracted significant interest due to their maximized atom efficiency, structural precision, and unique catalytic properties. While the central metal atom in SACs serves as the primary active site, its catalytic behavior is strongly influenced by the surrounding environment. The local coordination sphere plays a critical role in modulating the electronic structure and reaction pathway, thereby governing the overall activity, selectivity, and stability of SACs. Consequently, precise regulation of this coordination environment through rational engineering strategies is essential for optimizing the performance of SACs. Despite growing interest, comprehensive reviews systematically analyzing local coordination environments and metal‐support interactions via electronic descriptors remain scarce, unlike synthesis/application‐focused ones. In this review article, we provide an atomic‐level, descriptor‐driven analysis of coordination effects on the electrocatalytic performance of SACs, emphasizing the synergistic interplay between central metal atoms and their coordination spheres in activating key intermediates in reactions such as water splitting and oxygen/carbon dioxide reduction. We also highlight the unique features of advanced synthetic strategies for SACs, alongside the critical role of spectroscopic and microscopic techniques in elucidating structure–activity relationships, with particular focus on integrating operando investigations and theoretical calculations. Finally, recent advances, remaining challenges, and future opportunities in coordination‐regulated SAC electrocatalysis are discussed.

## Graphical abstract

This review summarizes atomic‐level design strategies for single‐atom catalysts, emphasizing modulation of the local coordination environment, active‐site geometry, and metal‐support interactions to optimize catalytic activity and stability in electrocatalytic energy conversion applications.

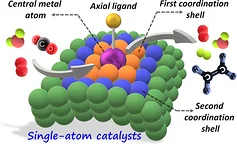

## 1 Introduction

The rapid depletion of fossil fuels and the substantial release of greenhouse gases have posed severe environmental and climate‐related challenges for modern society [1, 2]. To address these issues, the integration of electrochemical technologies with renewable energy sources such as solar and wind has emerged as a promising strategy to reduce dependence on fossil fuels. These sustainable systems have received increasing attention for applications in energy storage, artificial photosynthesis, and environmental remediation, owing to their ability to function without hazardous chemicals. Despite their potential, the development of efficient electrochemical processes remains a significant challenge, particularly for key reactions such as the oxygen evolution reaction (OER), hydrogen evolution reaction (HER), oxygen reduction reaction (ORR), and carbon dioxide reduction reaction (CO_2_RR) [3, 4, 5]. Central to the advancement of these systems are electrocatalysts, which are vital in lowering the energy barriers of these reactions and enabling enhanced activity, selectivity, and long‐term stability.

Supported metal nanoparticles (NPs) have long served as key components in heterogeneous catalysis, enabling a wide range of industrial chemical transformations [6]. However, conventional metal NPs often suffer from low atom utilization efficiencies, as only the surface atoms actively participate in catalytic reactions, while the bulk atoms remain catalytically inactive [7, 8]. This limitation not only reduces catalytic efficiency but also increases material costs, particularly for noble metals. In recent years, single‐atom catalysts (SACs) have emerged as a transformative class of catalytic materials, wherein individual metal atoms are atomically dispersed on diverse solid supports, including carbon materials, metals, metal oxides, and metal dichalcogenides [9, 10]. SACs offer several key advantages for electrocatalytic applications: (i) maximal atom utilization due to the complete exposure of active metal sites; (ii) the strong metal‐support interactions that modulate the electronic structure of the active sites, thereby enhancing the activation of reactant species and improving catalytic activity and selectivity; and (iii) the structurally well‐defined and uniform active sites enable more precise mechanistic studies, thus bridging the gap between homogeneous and heterogeneous catalysis [11, 12, 13]. Further, SACs offer high intrinsic activity and uniform active sites that can suppress side reactions. SACs can be broadly classified based on their coordination environments and stabilization strategies, including heteroatom‐coordinated sites, defect‐stabilized sites, and support‐interaction‐driven configurations. Such classification provides a useful basis for understanding how different coordination motifs influence catalytic behavior. Given these attributes, the rational design and development of SACs present significant opportunities for advancing high‐performance electrocatalysts and enabling next‐generation electrochemical energy conversion technologies.

In SACs, the central metal atoms are typically anchored to supports through covalent or ionic coordination bonds with surrounding atoms. Hence, the physicochemical properties of a single metal site in SACs are highly dependent on their local coordination environment, including coordination composition and coordination number (CN) [14, 15]. These factors critically influence the electronic structure by affecting orbital overlap and electron transfer, thereby modulating the interactions between the central metal atom, adsorbates, and the support. Therefore, rational local coordination engineering is essential for tailoring the catalytic activity, selectivity, and stability of SACs, establishing it as a core strategy in SAC development. The local coordination environment, defined as the first‐shell ligands (e.g., N/O/C, CN 2–6), second‐shell modifiers, neighboring defects, and proximal metal sites, fundamentally dictates SAC descriptors such as d‐band center, charge density, and spin‐state, modulating adsorption free energy of intermediates. On the other hand, the high surface free energy of isolated metal atoms makes them thermodynamically prone to aggregation into NPs, particularly under harsh reaction conditions, limiting long‐term stability and practical applicability [16]. The metal‐support interactions (MSIs), often ionic in nature, are key to stabilizing these single metal sites by inducing electronic perturbations that influence both stability and catalytic performance [17, 18, 19, 20]. While coordination environments define the immediate bonding configuration, MSIs extend beyond the first coordination shell and contribute to the stabilization and electronic modulation of active sites. Thus, the local coordination structure, together with MSIs, plays a decisive role in both the activity and stability of SACs, fundamentally distinguishing them from NPs‐based catalysts, whose performance is mainly governed by particle size, composition, and morphology. Over the past few years, numerous review articles have addressed various aspects of SACs, including synthetic methodologies, structural characterization techniques, and their applications in diverse electrocatalytic reactions [9, 20, 21, 22, 23, 24, 25, 26]. Meanwhile, advances in microscopic and spectroscopic techniques, alongside density functional theory calculations, have enabled in‐depth investigations into chemical environment‐activity relationships and underlying catalytic mechanisms [7, 17, 19]. However, despite this progress, most existing reviews predominantly focus on synthesis strategies or reaction‐specific performances, with limited emphasis on establishing a unified, descriptor‐level understanding that directly links coordination environments to catalytic activity, selectivity, and stability. A systematic framework that connects local coordination structures with key electronic descriptors and catalytic outcomes remains insufficiently developed.

In this context, the present review article aims to establish a descriptor‐centric perspective for understanding SAC electrocatalysis. Unlike prior reports, we focus on how modulation of local coordination environments and MSIs governs fundamental electronic descriptors such as d‐band center, charge distribution, and spin state, and how these, in turn, dictate adsorption energetics and reaction pathways in key electrocatalytic processes, including OER, HER, ORR, and CO_2_RR [27, 28]. We begin by outlining advanced synthetic strategies, not as isolated methodologies but as tools to construct specific coordination environments. This is followed by a discussion on how these coordination motifs influence electronic descriptors, providing the fundamental basis for rational catalyst design. Subsequently, we highlight how these coordination engineering approaches influence electrocatalytic performance across key reactions, with emphasis on structure–descriptor–activity relationships. Finally, we highlight critical challenges and future directions toward the rational and scalable design of next‐generation SACs.

## 2 Overview of SACs

Over the past few decades, catalysis has underpinned a vast array of chemical processes that constitute the foundation of modern industry, enabling the efficient synthesis of fuels, pharmaceuticals, polymers, and fine chemicals [6, 29]. By lowering activation energy barriers and directing reaction pathways, catalysts significantly enhance reaction rates and selectivity while minimizing energy consumption and waste generation. Currently, about 90% of industrial processes rely on two broad classes of catalysts: homogeneous and heterogeneous, each offering distinct advantages and inherent limitations [11, 30]. Homogeneous catalysts, such as enzymatic systems, are molecular species, often organometallic complexes, uniformly dispersed within the reaction medium, such that both catalyst and reactants exist in the same phase. The primary strength of homogeneous catalysts lies in their ability to provide precise molecular‐level control. Through rational ligand design, their electronic and steric properties can be finely tuned, mechanistic pathways are typically well understood at the atomic scale, and uniformity in active sites ensures high selectivity and reproducibility [31]. However, homogeneous systems are associated with several drawbacks, such as product separation is often challenging and costly, particularly when the catalyst and product share similar solubility characteristics. Furthermore, homogeneous catalysts may exhibit sensitivity to air, moisture, or reaction byproducts, which can adversely affect their stability and operational lifetime [32].

In contrast, heterogeneous catalysts consist of active species anchored on solid supports, with the catalyst and reactants residing in different phases. Metal NPs represent a typical example. Heterogeneous catalysts account for over 80% of the industrial chemical market, primarily due to their superior stability and ease of separation [11]. Their structural versatility, ranging from nanoparticles and clusters to porous frameworks, allows for tuning of surface area, pore architecture, and metal–support interactions to meet specific catalytic requirements [33]. Despite these advantages, heterogeneous catalysts frequently lack uniformity, as both their active sites and surface structures are inherently complex, making it difficult to elucidate detailed catalytic mechanisms. Moreover, under harsh reaction conditions, active sites in heterogeneous catalysts may undergo sintering, leaching, or poisoning, leading to gradual deactivation. For instance, the surfaces of metal NPs are composed of a diverse array of metal atoms in various spatial configurations, such as edges, faces, vertices, and corners, each corresponding to a distinct local coordination environment and exhibiting significantly different catalytic behaviors [34, 35, 36, 37, 38, 39, 40]. In addition, surface defects such as vacancies, stacking faults, and grain boundaries further influence catalytic performance [41, 42, 43]. Given that synthesizing NPs with identical distributions of active sites is practically unfeasible, accurate comparative evaluation of catalytic performance between different nanoparticles remains a considerable challenge.

Given the limitations of conventional catalysts, there is a growing interest in developing systems that combine the advantages of both homogeneous and heterogeneous catalysis. This has driven efforts to downscale metal NPs to the atomic level, leading to the emergence of SACs as illustrated in Figure 1 [7, 9, 10, 12, 44, 45, 46, 47, 48, 49, 50]. On the one hand, the reactivity of SACs can be finely tuned by modifying the local coordination environment of the active sites. On the other hand, the maximized atomic utilization improves mass activity while potentially reducing production costs. The origin of SACs dates back to 1999, when Asakura and coworkers demonstrated that atomically dispersed Pt ions supported on MgO could catalyze propane combustion [51]. The absence of Pt‐Pt bonds, confirming the absence of Pt NPs, was established using extended x‐ray absorption fine structure (EXAFS) analysis. In 2003, Fu et al. demonstrated that the Pt/Au single atoms on the CeO_2_ substrate are the essential active center rather than the metallic NPs [52]. With the progression of advanced spectroscopic and microscopic characterization techniques, SACs entered a period of accelerated development [53]. A major milestone was achieved in 2011 when Zhang and coworkers introduced the first modern definition of SACs using a Pt_1_/FeO_x_ proof‐of‐concept model [54]. By integrating in situ spectroscopic techniques with density functional theory calculations, they elucidated the relationship between the electronic structure of isolated Pt atoms and their catalytic performance. High‐angle annular dark‐field scanning transmission electron microscopy (HAADF‐STEM) played a crucial role in directly visualizing Pt single atoms anchored on the support surface. Subsequently, carbon‐based materials emerged as attractive supports for SACs. In 2015, a significant breakthrough was achieved with the successful construction of Co‐N_4_ coordination units on nitrogen‐doped carbon frameworks (Co‐N‐C), representing a shift toward coordination engineering as a core strategy for SAC synthesis and performance modulation [55, 56, 57, 58, 59].

**FIGURE 1:**
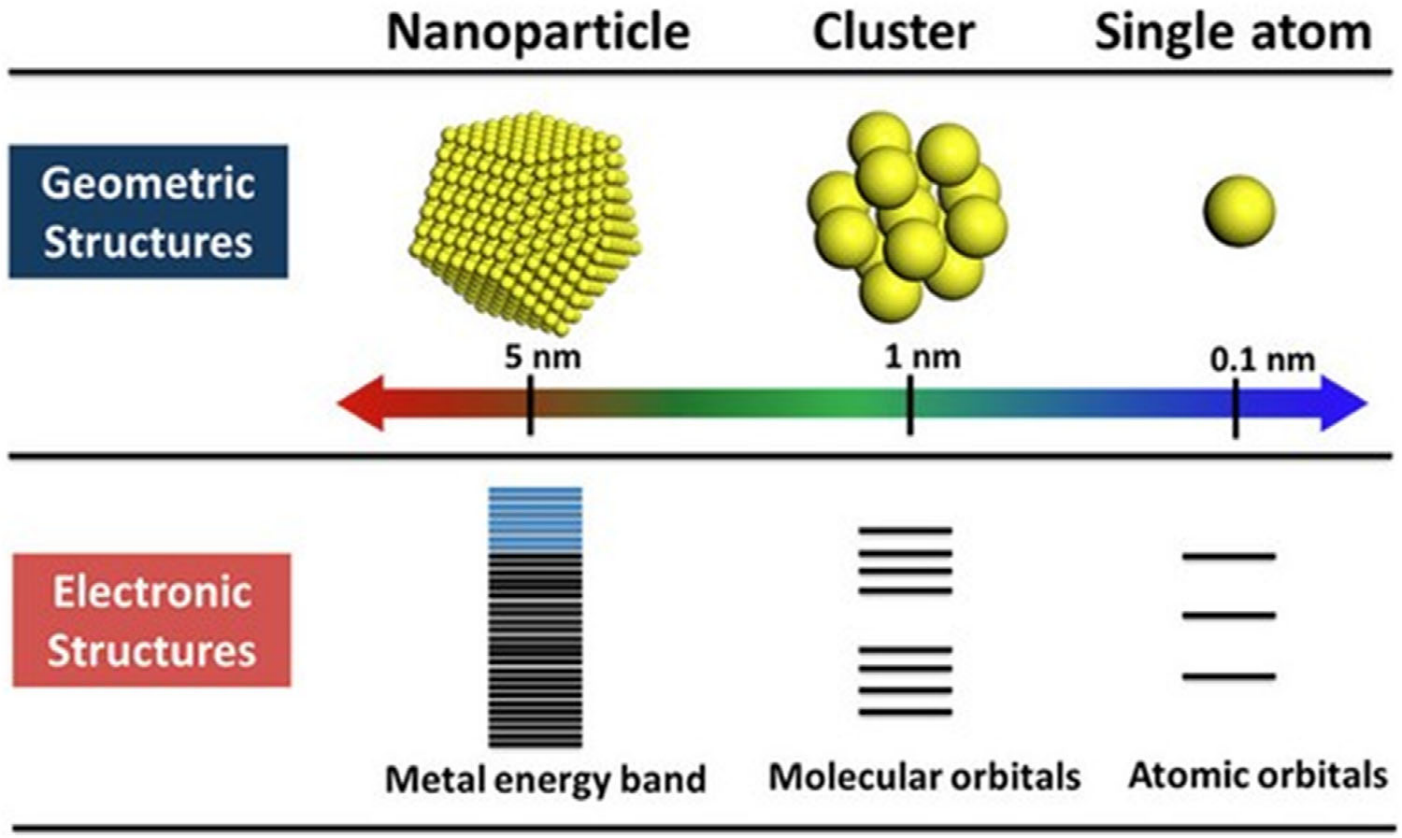
SACs represent the ultimate downsizing of metal nanoparticles, offering maximal atom utilization with uniform active sites. Figure adapted with permission from ref [12]. Copyright 2020, Wiley‐VCH.

Since then, SACs have garnered widespread interest across diverse fields of catalysis. A central focus has been the precise characterization and control of the local coordination structure, which is crucial for understanding and optimizing catalytic behavior. The development of sophisticated in situ/operando techniques such as x‐ray absorption spectroscopy (XAS), aberration‐corrected HAADF‐STEM, diffuse reflectance infrared Fourier transform spectroscopy (DRIFTS) with CO chemisorption, isotope labeling, etc., along with advanced computational modeling, has provided unprecedented insights into the role of local chemical environments in SACs [60, 61, 62, 63, 64]. Today, a wide variety of coordination engineering‐based strategies have been reported, enabling fine‐tuned design of highly active, selective, and stable SACs [65, 66]. The next section will focus on summarizing the state‐of‐the‐art in SAC synthesis strategies, emphasizing their unique features and the technical challenges that remain.

## 3 Synthetic Strategies of SACs

A major challenge in the early development of SACs was the inherent tendency of isolated metal atoms to migrate and aggregate into clusters or nanoparticles during high‐temperature treatments, driven by their high surface energy [9, 67, 68, 69]. This issue significantly limited the stability and scalability of SACs. However, substantial progress has been made through the development of advanced synthetic strategies capable of achieving atomically dispersed metal species with appreciable loading levels [21, 70, 71, 72, 73, 74]. In this section, we present an overview of the underlying principles and distinctive features of several recent synthetic methodologies for SACs. These include defect engineering, pyrolysis‐based methods, electrochemical deposition, galvanic replacement, spatial confinement, atomic layer deposition, and photochemical approaches. Particular emphasis is placed on the innovative aspects and current limitations of these approaches, especially regarding their effectiveness in anchoring isolated atoms at higher densities and maintaining the stability of active sites under extended catalytic conditions.

Importantly, rather than treating synthesis as an isolated aspect, these approaches are discussed here as tools to construct well‐defined coordination environments. Each method offers distinct control over coordination number, ligand identity, defect density, and metal‐support interactions, which ultimately govern key electronic descriptors such as d‐band center, charge distribution, and spin state, as elaborated in Section 4. This perspective establishes a direct link between synthetic design and catalytic function, forming the basis for the coordination engineering strategies discussed in subsequent sections.

### 3.1 Defect Engineering

Defect engineering represents a robust approach for the stabilization and synthesis of SACs by utilizing defect/vacancy‐rich support surfaces to trap and immobilize isolated metal atoms (Figure 2a,b) [75, 76, 77]. These defect sites, including vacancies (e.g., oxygen, nitrogen, sulfur, and carbon), dislocations, step edges, and grain boundaries, offer thermodynamically favorable coordination environments that strongly interact with metal atoms through mechanisms such as charge transfer or hybrid orbital overlap [78, 79]. Such interactions prevent metal atom migration and aggregation, thereby maintaining their atomic dispersion. Ideal support materials for SAC stabilization are those that require minimal pre‐treatment (e.g., thermal or chemical etching) and intrinsically exhibit strong metal‐support interactions. Materials such as graphene, metal oxides, hydroxides, and MXenes are among the most widely used candidates due to their tunable defect chemistry [9, 19, 77, 78, 80]. The nature and density of these defects play a crucial role in determining both the metal loading and the stability of the SACs [81]. Atomically dispersed metals anchored at defect sites benefit from localized electronic environments, which not only stabilize the metal centers but also influence their catalytic activity by modulating charge density and adsorption properties. Furthermore, defect engineering allows fine‐tuning of the coordination structure, which is critical for tailoring the catalytic properties of SACs toward specific electrochemical reactions [82, 83]. These defect‐induced coordination environments typically lead to localized charge redistribution and modified d‐band centers at the metal sites, directly influencing adsorption energetics and catalytic activity.

**FIGURE 2:**
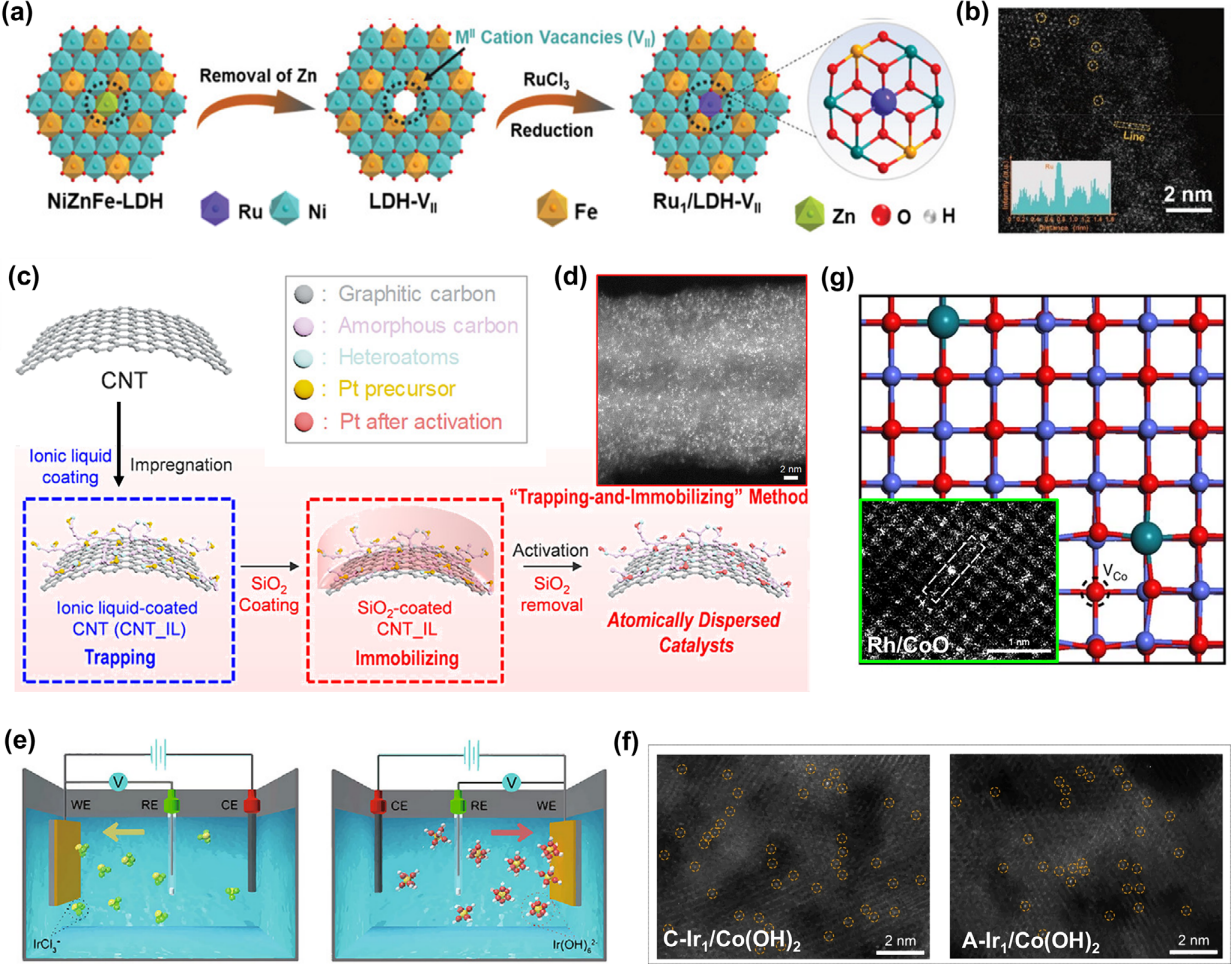
(a) Schematic illustration of the defect engineering strategy and (b) aberration‐corrected HAADF‐STEM image for Ru_1_/LDH‐V_II_. Reproduced with permission from ref [75]. Copyright 2022, Wiley‐VCH. (c) Schematic representation of the trapping‐and‐immobilization approach used to synthesize atomically dispersed noble metal catalysts and (d) HAADF‐STEM image of Pt SACs on support. Reproduced with permission from ref [98]. Copyright 2020, American Chemical Society. (e) Illustration of electrochemical deposition of Ir species on the cathode (left)/anode (right) and (f) HAADF‐STEM images of C‐Ir_1_/Co(OH)_2_ and A‐Ir_1_/Co(OH)_2_. Reproduced with permission from ref [99]. Copyright 2020, Springer Nature. (g) Galvanic replacement route for the synthesis of the Rh/CoO model system. The inset shows the HAADF‐STEM image. Reproduced with permission from ref [106]. Copyright 2016, Springer Nature.

Despite its promise, precise and periodic control over defect distribution remains a major challenge. Current techniques, such as plasma treatment, ion bombardment, chemical etching, and high‐temperature pyrolysis under reductive conditions, can introduce defects but often lead to their random distribution [21, 75, 84]. Addressing this limitation requires continued advancement in synthetic methodologies and fundamental understanding of defect formation, which will be vital for achieving the rational design of SACs with atomic precision. Integrating defect engineering with in situ/operando characterization and theoretical modeling may provide deeper insights into the structure–activity relationship and unlock new strategies for controllable SAC synthesis.

### 3.2 High Temperature Pyrolysis Process

High‐temperature pyrolysis has become a widely utilized and scalable approach for synthesizing SACs with tailored structural and chemical properties. However, a major challenge associated with this method is the sintering and aggregation of isolated metal atoms into clusters or nanoparticles due to enhanced atomic mobility at elevated temperatures, which can significantly impair catalytic performance [85]. To mitigate these issues, thermally stable supports capable of anchoring mobile metal atoms are essential to inhibit migration and aggregation. Commonly used supports include nitrogen‐doped carbons, metal‐organic frameworks (MOFs), polymeric matrices, and so forth, which are rich in heteroatoms and exhibit high surface areas [59, 82, 86, 87, 88, 89]. During pyrolysis under inert or reductive atmospheres, these organic frameworks decompose into conductive carbon matrices, while simultaneously forming strong metal‐support interactions such as coordination with nitrogen or encapsulation within porphyrin‐like moieties (e.g., M‐N_4_ structures), which help stabilize the isolated metal atoms and enhance their sintering resistance [90, 91, 92, 93]. An important advancement in this context was introduced by Datye and coworkers in 2016, who developed a high‐temperature atom trapping strategy. By thermally treating Pt/La‐Al_2_O_3_ in the presence of ceria powder, they demonstrated that Pt NPs could disintegrate into single atoms and become immobilized on ceria nanorods [67]. Around the same time, Corma and colleagues reported a reversible redispersion of Pt NPs into atomically dispersed Pt sites under oxidative conditions, with reformation under reducing atmospheres [94]. In parallel, porous carbon supports with tunable textural and electronic features are being explored to regulate the local coordination environment and mass transport properties of SACs [95, 96, 97]. Recently, a generalized “trapping‐and‐immobilizing” strategy has enabled the synthesis of noble‐metal SACs via precursor trapping on heteroatom‐doped carbon supports, followed by high‐temperature treatment under a protective SiO_2_ layer (Figure 2c,d) [98]. The thermally evolved coordination structures are often accompanied by shifts in the d‐band center and metal oxidation state, which play a decisive role in tuning catalytic performance. Despite such advances, structural degradation, such as partial pore collapse and surface area reduction, remains a challenge. Thus, the use of hard templates with intrinsic porosity, like zeolitic imidazolate frameworks, offers a promising solution to preserve structural integrity and enhance mass transport during high‐temperature SAC synthesis.

### 3.3 Electrochemical Deposition

Electrochemical deposition has emerged as a robust and versatile bottom‐up strategy for the synthesis and stabilization of SACs, offering precise control over atom loading, site coordination, and deposition selectivity [99]. Typically conducted in a standard three‐electrode configuration, this method employs electrochemical techniques such as linear sweep voltammetry, chronoamperometry, and potential‐cycling to anchor metal ions onto conductive supports [78, 99, 100, 101, 102, 103]. Unlike traditional chemical or thermal synthesis methods, electrochemical deposition enables site‐specific nucleation of metal atoms by tuning electrochemical parameters such as applied potential, current density, pH, and electrolyte composition. By carefully optimizing metal precursors and supports, isolated metal atoms such as Pt, Ir, Ru, Ag, Fe, Co, Ni, and so forth can be successfully stabilized on supports via cathodic or anodic deposition (Figure 2e,f) [99]. Interestingly, anodic and cathodic depositions can yield different electronic structures for the same metal, resulting in distinct catalytic properties. Importantly, the electrochemical control over deposition conditions enables tuning of the local coordination environment and metal valence, thereby modulating charge distribution and adsorption properties at the active sites.

Further studies have demonstrated the use of potential‐cycling deposition to prepare large‐area, binder‐free SACs, including Pt single atoms on CoP nanotubes for efficient hydrogen evolution in neutral media [102]. Electrochemical activation strategy has also enabled the transformation of encapsulated metal nanoparticles, such as Ni within graphitic carbon, into atomically dispersed species under acidic conditions [104]. The electrochemical activation strategy has been extended to generate Co and Pt SACs on N‐doped defective carbons, showing high activity for oxygen reduction reactions [105]. Despite its strengths, the electrochemical approach is challenged by the limited metal loading capacity, constrained by the supersaturation threshold of the substrate. Exceeding this threshold can lead to atom aggregation and the formation of clusters. Therefore, the choice of support material with high metal affinity and strong stabilization capacity is crucial to prevent agglomeration and ensure catalyst durability.

### 3.4 Galvanic Replacements

Galvanic replacement is a widely utilized electrochemical strategy for the synthesis of SACs, based on spontaneous redox reactions driven by differences in the standard reduction potentials of two metals (Figure 2g) [106]. In this process, a less noble metal serving as the sacrificial template undergoes oxidation, dissolving into solution and generating atomic vacancies within the host lattice. Concurrently, metal ions from a more noble precursor with a higher reduction potential are reduced and deposited onto the template, often anchoring at the created vacancies [49, 107]. This atomically resolved replacement mechanism allows for the selective incorporation of noble metal atoms at specific lattice sites, enabling the formation of highly dispersed single‐atom active centers with minimal aggregation. The galvanic replacement approach allows fine control over the loading, dispersion, and coordination environment of single atoms by adjusting parameters such as metal precursor concentration, reaction time, temperature, and substrate morphology [108]. These capabilities have been effectively demonstrated in the preparation of single‐atom alloys, including Pt‐Cu, Ru‐Ni, and Rh‐Co systems, where the noble metal atoms are embedded within a less noble metal host, exhibiting synergistic effects that enhance catalytic activity and selectivity [106, 109, 110]. Despite its advantages, galvanic replacement does present inherent limitations. A critical requirement is a sufficient difference in reduction potential between the sacrificial and depositing metals to drive the redox process. Additionally, the method often yields catalysts with relatively low metal loading and heterogeneous coordination structures, which can compromise their catalytic efficiency, selectivity, and durability under operational conditions [111]. Therefore, the galvanic replacement technique is particularly suited for synthesizing noble‐metal‐based SACs, where it serves the dual purpose of minimizing precious metal usage and exploiting their superior chemical stability. This atom‐by‐atom replacement process can generate unique coordination environments within host lattices, often leading to modified electronic structures, including d‐band shifts and charge redistribution at the active sites.

### 3.5 Spatial Confinements

Spatial confinement is an innovative and highly effective strategy for the synthesis of SACs, which aims to isolate individual metal atoms within well‐defined nanoscale environments to prevent aggregation and enhance catalytic performance (Figure 3a,b) [112, 113]. Unlike techniques that require capturing agents or stabilizing ligands, spatial confinement uses the intrinsic architecture of porous frameworks such as zeolites, MOFs, and covalent organic frameworks (COFs) to physically control atomic diffusion, thereby preventing metal atom migration and aggregation under thermal or reactive conditions [21, 112, 114, 115]. The confinement effect also enables strong interactions between the isolated metal atoms and the host matrix, which can modify the electronic structure and coordination environment of the metal centers, tailoring their catalytic properties. The synthesis typically involves the incorporation of metal precursors into sub‐nanometer pores or cages of the host matrix via wet‐chemical techniques, including impregnation, ion‐exchange, or co‐precipitation [54, 116, 117]. Subsequent thermal treatments result in the formation of atomically dispersed metal species anchored within the confined space. The confined coordination environments imposed by porous hosts can significantly alter the electronic structure of metal centers, including their charge state and orbital interactions, thereby influencing catalytic descriptors.

**FIGURE 3:**
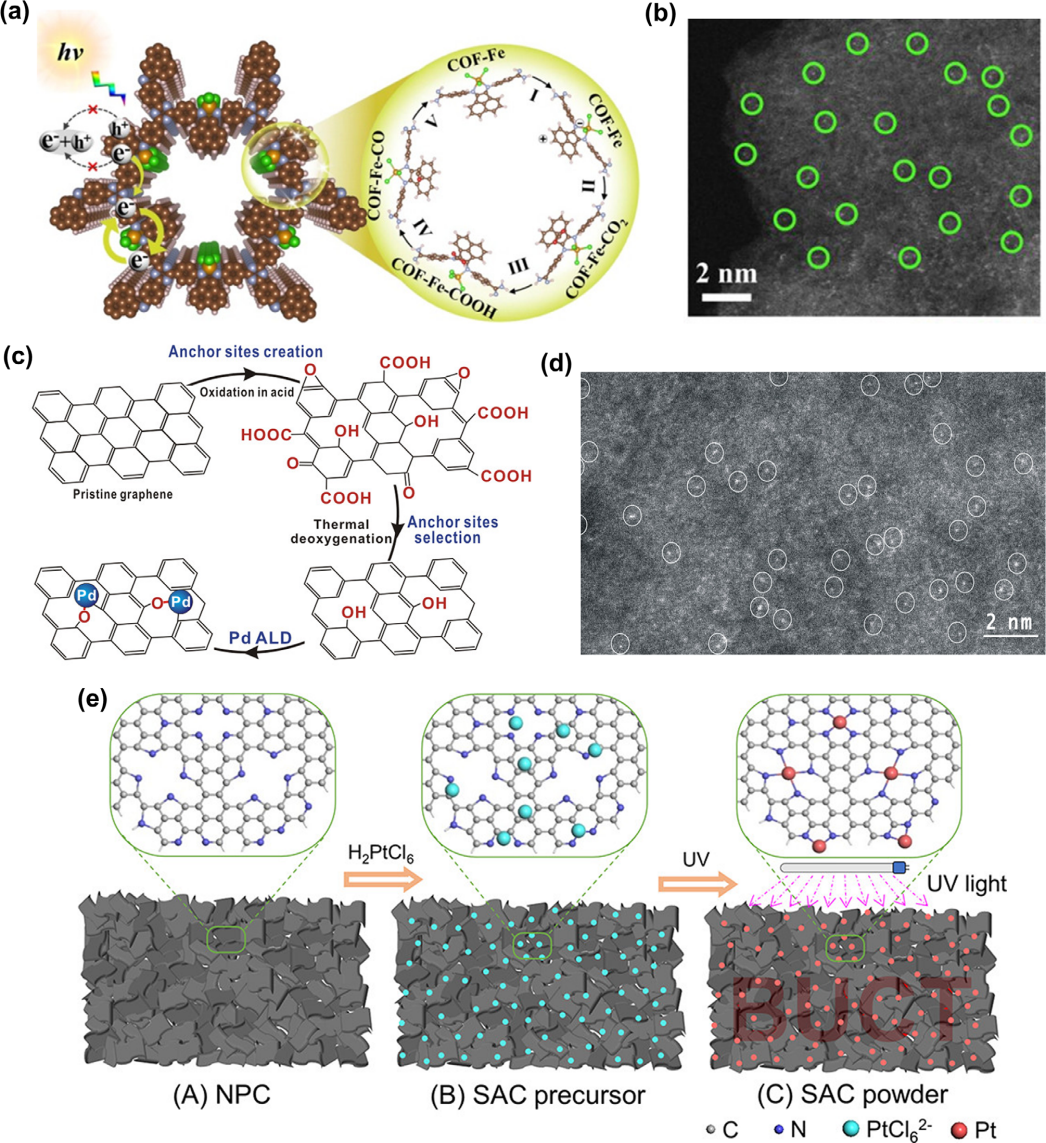
(a) Fe SAS/Tr‐COFs synthesized via spatial confinement method toward photocatalytic CO_2_RR and (b) aberration‐corrected HAADF–STEM image of 1.1 wt % Fe SAS/Tr‐COFs. Reproduced with permission from ref [112]. Copyright 2022, American Chemical Society. (c) Schematic illustration showing the synthetic strategy for Pd_1_/graphene catalyst involving the creation and selection of anchoring sites, followed by ALD of Pd on pristine graphene and (d) HAADF‐STEM image of Pd_1_/graphene at high magnifications. Reproduced with permission from ref [123]. Copyright 2015, American Chemical Society. (e) Schematic depiction of the synthesis of Pt_1_/NPC catalyst via a photochemical reduction method. Reproduced with permission from ref [129]. Copyright 2018, American Chemical Society.

A notable advantage of spatial confinement is its ability to impart uniform and well‐defined coordination geometries to the active sites, as observed in SACs embedded within crystalline matrices like zeolites and MOFs [118]. However, due to the thermodynamic limitations of precursor encapsulation, incomplete filling of pore spaces can lead to relatively low metal loadings [119]. To mitigate this, extending treatment durations or applying external agitation such as stirring or shaking during synthesis can improve precursor diffusion and uptake.

### 3.6 Atomic Layer Deposition

Atomic Layer Deposition (ALD) is a precise and controllable thin‐film deposition technique that has emerged as a powerful method for the large‐scale synthesis of stable SACs [120]. ALD operates through sequential, self‐limiting surface reactions that allow for the deposition of materials with atomic‐level control over thickness, composition, and dispersion [21, 121]. In the context of SACs, ALD enables the uniform deposition of metal precursors onto high‐surface‐area supports, resulting in isolated metal atoms anchored at well‐defined sites such as oxygen vacancies, heteroatoms, or defect sites. In a standard ALD cycle, a volatile metal precursor reacts with surface functional groups, followed by a purging step and subsequent exposure to a co‐reactant or oxidant to complete the surface reaction. Repeating these cycles allows fine‐tuning of the metal loading while preserving atomic dispersion. A pioneering example was demonstrated by Sun and coworkers in 2013, where (methylcyclopentadienyl)‐trimethylplatinum and oxygen were employed as precursors to deposit Pt atoms on graphene nanosheets. By modulating the number of ALD cycles, they achieved precise control over the Pt loading, size, and dispersion, ultimately achieving atomically dispersed Pt after 50 cycles [122]. Similarly, Pd atoms have also been atomically deposited onto oxygen‐functionalized graphene surfaces using this technique (Figure 3c,d) [123]. To improve mass transport and maximize single‐atom exposure, ALD has been further applied to vertically aligned N‐doped graphene nanomesh arrays for Pt and Pd deposition [124]. Such atomic‐level control over coordination geometry enables systematic tuning of electronic descriptors, including d‐band center and metal‐support charge transfer, which are critical for catalytic activity. Despite its limitations in scalability and cost, ALD remains a vital technique for the rational design of SACs due to its unmatched control over atomic‐level structure and composition.

### 3.7 Photochemical Method

The photochemical method has emerged as a promising approach for the synthesis and stabilization of SACs, offering precise control over metal dispersion and oxidation states under mild conditions [125]. This technique utilizes light‐induced chemical reactions to reduce metal precursors and anchor single metal atoms onto suitable supports. Upon light irradiation, photoexcited electrons and holes are generated in the support, which can facilitate the reduction of metal ions to atomic species and their immobilization at defect sites, heteroatoms, or surface vacancies [126]. Unlike conventional thermal methods, photochemical routes operate under ambient temperature and pressure, significantly reducing the tendency for metal atom aggregation and enabling higher dispersion of single atoms. Additionally, the use of light as a stimulus allows for spatial and temporal control over nucleation and growth processes, making it possible to finely tune metal loading and dispersion [125, 127]. Notably, this technique enables the fabrication of SACs with metal loadings up to three times higher than those obtained via traditional sintering‐based methods [65]. A pioneering example was reported in 2013 by Flytzani‐Stephanopoulos and coworkers, who stabilized Au single atoms on TiO_x_ through UV‐assisted reduction in the presence of ethanol as a charge scavenger.

Even after the removal of excess Au nanoparticles via cyanide leaching, atomically dispersed Au remained active and anchored [128]. To further suppress metal nucleation, an innovative iced‐photochemical reduction technique was developed, wherein H_2_PtCl_6_ solutions were frozen and irradiated with UV light, enabling Pt single atoms to be confined within the ice lattice and anchored on various supports [74]. Additionally, a modified solid‐phase photochemical reduction approach has been used to prepare well‐defined Pt SACs on N‐doped porous carbon, achieving loadings as high as 3.8 wt% without forming nanoparticles (Figure 3e) [129]. These advances highlight the photochemical method as a green, scalable, and highly effective route for designing high‐performance SACs. The mild synthesis conditions can preserve low‐coordination environments and distinct electronic states, enabling fine control over charge density and spin configuration of isolated metal sites.

Although these synthetic approaches enable control over atomic dispersion and coordination structures, the catalytic performance of SACs is ultimately governed by how these local coordination environments influence their electronic structure and adsorption properties. Therefore, establishing a descriptor‐based understanding of structure–property relationships is essential, as discussed in the following section.

## 4 Coordination Environment Effects on Electronic Descriptors

The synthetic approaches discussed in Section 3 do not merely enable the formation of SACs; more importantly, they define the local coordination environments surrounding the isolated metal centers, which ultimately govern catalytic performance. Establishing a clear understanding of how these coordination environments influence electronic structure is, therefore, essential before discussing specific modulation strategies.

*What does coordination control?* The local coordination environment typically comprises the first coordination shell‐metal‐ligand bonds within ∼3 Å, with coordination numbers generally ranging from 2 to 6, and the second coordination sphere, which can influence the electronic structure through indirect interactions. This is distinct from MSIs, which often involve longer‐range effects like charge transfer across the support surface. These structural features regulate key electronic descriptors, including the d‐band center, metal charge/oxidation state, and spin states, all of which dictate the adsorption and activation of reaction intermediates as shown in Figure 4.

- *d‐band center position*: The position of metal d‐orbitals (d‐band center) relative to the Fermi level plays a central role in determining adsorption strength and controls how strongly reaction intermediates (H*, OH*, OOH*, CO*, etc.) bind to the surface. A shift of the d‐band center toward the Fermi level generally strengthens the binding of intermediates, whereas a downward shift weakens adsorption [130]. Consequently, catalytic activity follows a Sabatier‐type behavior, where optimal performance is achieved at intermediate binding strength. For example, changing the coordination environment of metal centers (e.g., from M‐N_4_ to M‐O_4_) can upshift the d‐band center close to the Fermi level, thereby enhancing the adsorption of key intermediates such as COOH* in CO_2_ reduction, while excessive binding may hinder product desorption [131].
- *Metal charge/oxidation state: *Modulation of the metal charge state significantly influences catalytic behavior. Electron‐withdrawing ligands such as nitrogen tend to increase the oxidation state of the central metal, whereas defect sites (e.g., oxygen vacancies) or electron‐rich supports can donate charge, altering adsorption energetics [9, 59]. For instance, charge transfer from defect‐rich oxide supports to anchored metal atoms can tune intermediate binding, thereby facilitating reaction kinetics [17]. Similarly, second‐shell heteroatoms (S/P) can induce electronic polarization without directly altering the primary coordination structure, offering an additional degree of control [132].
- *Spin‐state: *Spin‐state regulation represents another important yet often overlooked descriptor. Variations in coordination geometry and ligand field strength can alter the spin configuration of transition metal centers, thereby influencing orbital occupancy and overlap with adsorbates. Weak field ligands often stabilize high‐spin states, which can enhance reactivity toward bond activation steps, although sometimes at the expense of stability [133].

**FIGURE 4:**
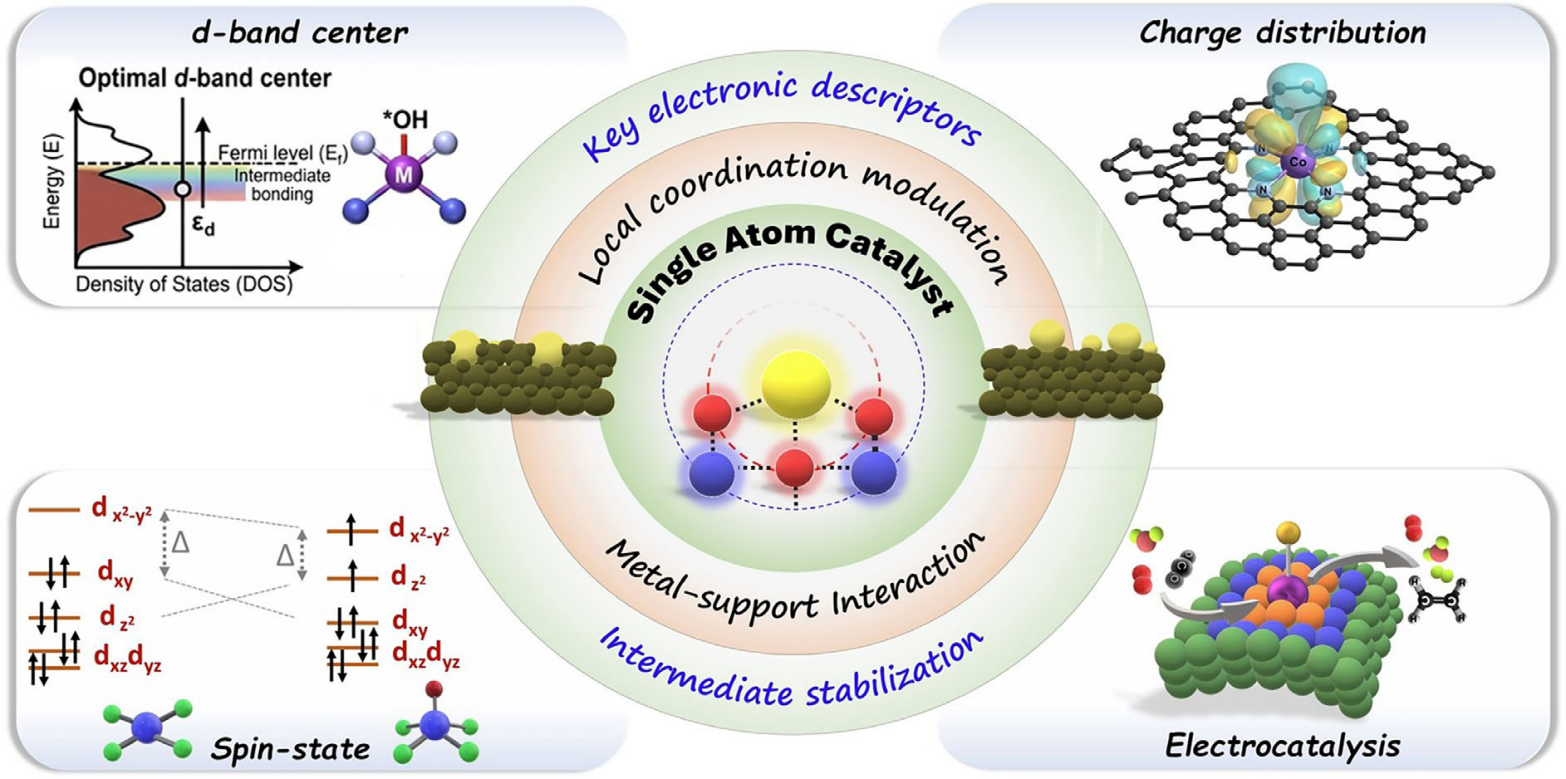
Schematic illustration of how local coordination modulation and metal–support interactions govern key electronic descriptors (d‐band center, charge distribution, and spin state), thereby dictating intermediate stabilization and overall electrocatalytic performance of SACs.

Importantly, the adsorption energies of key intermediates are not independent but are often linked through linear scaling relationships, which impose intrinsic limitations on catalytic performance. Rational coordination engineering provides a pathway to tune these scaling relations by selectively stabilizing specific intermediates, thereby lowering reaction barriers and improving efficiency. In addition, strong MSIs can stabilize isolated metal sites under operating conditions while simultaneously modulating charge transfer, further influencing catalytic activity and durability.

*How do synthesis approaches create these effects*? From a synthetic perspective, different preparation methods inherently create distinct coordination environments and thus different electronic structures. For example, high‐temperature pyrolysis often regulates coordination numbers and introduces defects, leading to tuned d‐band centers and enhanced reactivity in reactions such as CO_2_ reduction [92, 134]. Defect engineering introduces charge‐rich sites that modulate metal oxidation states and adsorption properties, while atomic layer deposition enables precise control over coordination geometry, allowing systematic tuning of catalytic behavior [76, 124]. Photochemical methods, in contrast, can stabilize low‐coordination sites at relatively high loadings without inducing aggregation, thereby preserving highly active configurations.

It is also important to recognize that these coordination environments are not always static under reaction conditions. *Operando* studies have revealed dynamic restructuring of active sites, such as ligand exchange or coordination number changes, which can further influence catalytic performance. Such dynamic behavior underscores the need for in situ and operando characterization to accurately capture structure–activity relationships.

Overall, the coordination environment in SACs serves as a fundamental handle for tuning electronic descriptors and adsorption energetics, thereby dictating catalytic activity, selectivity, and stability. This descriptor‐based understanding provides a rational framework for designing advanced SACs, forming the basis for the coordination modulation strategies discussed in the following section.

## 5 Local Coordination Modulation Strategies in SACs

As discussed in Section 4, the catalytic performance of SACs is intrinsically governed by their local coordination environments through modulation of key electronic descriptors, including the d‐band center, charge distribution, spin state, and adsorption energetics of reaction intermediates. Therefore, rational manipulation of these coordination environments provides a direct and effective pathway to tailor catalytic performance. In this context, coordination engineering has emerged as a central design principle for advancing SACs beyond empirical optimization toward descriptor‐driven catalyst design. Unlike conventional heterogeneous catalysts, SACs consist of atomically dispersed metal centers stabilized by surrounding atoms within the support matrix, forming well‐defined coordination structures. However, under realistic catalytic conditions such as elevated temperatures or electrochemical potentials, these isolated atoms are prone to migration and aggregation due to their high surface energy, leading to catalyst deactivation. Hence, precise control over the local coordination environment is not only essential for tuning electronic structure and adsorption behavior but also for ensuring long‐term structural stability [135, 136]. Various strategies have been developed to modulate the coordination environment at the atomic scale, including engineering strong MSIs, employing high‐temperature treatments to induce controlled structural evolution, constructing synergistic dual‐atom motifs, introducing heteroatom dopants to tailor ligand fields, and integrating single atoms with nanoclusters or nanoparticles to exploit interfacial electronic effects. Each of these approaches alters the coordination number, ligand identity, and local electronic structure, thereby tuning the adsorption energies of key intermediates and influencing reaction pathways [20, 137]. The following subsections provide a detailed discussion of these strategies, highlighting their design principles, structure–property relationships, and implications for catalytic performance.

### 5.1 Electronic MSIs

In SACs, the isolated metal atoms are typically anchored to the support via covalent or ionic bonds, forming well‐defined coordination environments. As discussed in Section 4, MSIs play a critical role in modulating key electronic descriptors such as metal charge density, oxidation state, and d‐band center through long‐range charge transfer effects. Consequently, the strength and nature of MSIs directly determine the stability, electronic configuration, and catalytic reactivity of the active sites [20]. For example, Qiao et al. demonstrated the in situ stabilization of atomically dispersed Pt on Fe_2_O_3_ during methane oxidation, achieved through strong covalent bonding with surface Fe and O atoms, independent of support defects (Figure 5a) [138]. These strong MSIs facilitate charge redistribution between Pt (Bader charge: +1.43|e|) and the oxide support, stabilizing isolated Pt atoms and maintaining an optimal electronic structure for methane activation. As a result, the Pt SACs exhibited exceptional thermal stability and high methane combustion activity, with the reducibility of iron oxide playing a key role in tuning the electronic environment of Pt centers. In 2019, Lu and coworkers employed ALD to synthesize Pt single atoms on Co_3_O_4_, CeO_2_, ZrO_2_, and graphene (Figure 5b) [139]. The Pt L_3_‐edge XANES analysis revealed that Pt_1_/Co_3_O_4_ possessed a higher oxidation state (∼+4) and maximum 5d vacancy than other SACs, suggesting stronger MSI in Pt_1_/Co_3_O_4_ (Figure 5c). This upward shift in oxidation state reflects significant charge transfer from Pt to the support, which modifies the d‐band electronic structure and optimizes adsorption/desorption energetics. The Pt_1_/Co_3_O_4_ delivered outstanding catalytic activity and durability in ammonia borane dehydrogenation for room‐temperature hydrogen generation, without performance loss or aggregation (Figure 5d,e). Theoretical calculations further indicate that the MSI modulates the unoccupied 5d states of Pt single atoms, optimizing ammonia borane adsorption and promoting hydrogen desorption, thereby enhancing catalytic activity. It is worth noting that oxide supports, although highly effective in stabilizing isolated metal atoms via strong MSIs, often exhibit relatively low intrinsic electrical conductivity. This can impose limitations on interfacial charge transfer during electrocatalysis, thereby affecting overall reaction kinetics. To address this, strategies such as defect engineering (e.g., oxygen vacancy generation), heteroatom doping, and coupling with conductive carbon‐based substrates are commonly employed to enhance electronic conductivity while preserving the coordination environment of active sites [9, 17, 30]. Furthermore, Lu and coworkers also reported that Pd SACs on C_3_N_4_ and graphene exhibited markedly different hydrogenation activities toward acetylene and 1,3‐butadiene (Figure 5f) [140]. This difference was attributed to variations in Pd valence states arising from distinct MSIs. Specifically, Pd_1_/graphene exhibited a lower oxidation state compared to Pd_1_/C_3_N_4_, indicating higher electron density and a shifted d‐band center closer to the Fermi level. This electronic configuration strengthens the adsorption of hydrogenation intermediates, leading to enhanced catalytic activity.

**FIGURE 5:**
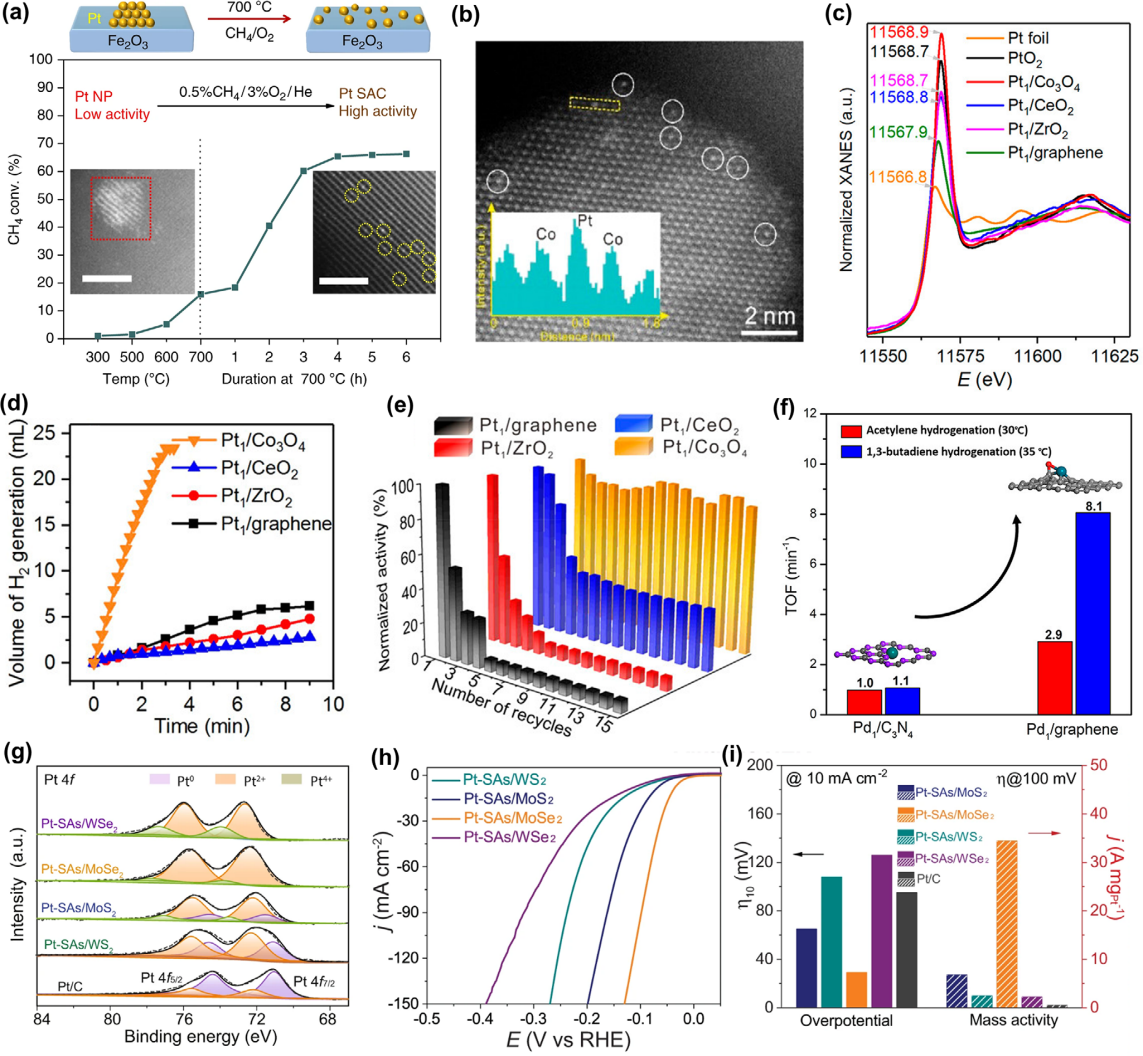
(a) In situ dynamic formation of Pt single atoms on Fe_2_O_3_ during methane oxidation, facilitated by strong covalent interactions between Pt, surface oxygen, and iron atoms. Reproduced with permission from ref [138]. Copyright 2019, Springer Nature. (b) Aberration‐corrected HAADF‐STEM image of atomically dispersed Pt species on Co_3_O_4_ (Pt_1_/Co_3_O_4_), (c) normalized Pt L_3_‐edge XANES spectra, (d) comparison of catalytic performance, and (e) recyclability tests in ammonia borane dehydrogenation over Pt_1_/Co_3_O_4_, Pt_1_/CeO_2_, Pt_1_/ZrO_2_, and Pt_1_/graphene. Reproduced with permission from ref [139]. Copyright 2019, American Chemical Society. (f) Turnover frequencies (TOFs) for Pd_1_/C_3_N_4_ and Pd_1_/graphene SACs in selective hydrogenation of acetylene and 1,3‐butadiene. Reproduced with permission from ref [140]. Copyright 2019, American Chemical Society. (g) Pt 4f XPS spectra of the Pt‐SAs/TMDs samples compared with commercial Pt/C, (h) HER LSV polarization curves of various Pt‐SAs/TMDs samples in alkaline electrolyte, and (i) summary of HER performance across Pt‐SAs/TMDs samples, showing overpotentials at 10 mA cm^−2^ (black arrow, left axis) and mass activities normalized to Pt loading (red arrow, right axis) at ‐100 mV versus RHE. Reproduced with permission from ref [141]. Copyright 2021, Springer Nature.

Recently, Chen et al. demonstrated that precise regulation of the Pt oxidation state through electronic MSIs with transition‐metal dichalcogenides (TMDs) can significantly influence alkaline HER performance [141]. XPS analysis of the Pt 4*f* region revealed that Pt species in Pt‐SAs/TMDs exhibit partial oxidation (Pt^δ+^: 0 to +4), arising from mutual electronic coupling between Pt atoms and the TMD supports (Figure 5g). This tunable oxidation state systematically shifts the d‐band center upward, thereby governing HER activity by modulating Pt‐H and Pt‐OH binding strengths. Among the studied systems, Pt‐SAs/MoSe_2_ exhibited the lowest overpotential at 10 mA cm^−2^ and the highest mass activity at an overpotential of 100 mV, outperforming all other Pt‐SAs/TMD catalysts (Figure 5h,i). Beyond electronic effects, tailoring the support morphology also proved effective in enhancing SAC performance. For instance, Pt single atoms anchored on zero‐dimensional onion‐like carbon showed lower HER overpotentials and superior mass activity compared to those on two‐dimensional graphene, despite both being synthesized via ALD [142]. This enhancement was attributed to the high curvature of onion‐like carbon, which induces a tip‐enhanced local electric field at the Pt sites, thereby increasing local proton concentration and accelerating HER kinetics. Overall, these studies clearly demonstrate that electronic MSIs serve as an effective handle to tune key descriptors such as oxidation state, d‐band structure, and adsorption energetics. Such modulation not only stabilizes isolated metal atoms but also optimizes catalytic pathways, underscoring the importance of rational MSI engineering in SAC design.

### 5.2 Coordination Modulation via Thermal Evolution

Thermal reconstruction treatments represent a powerful approach for modulating the local coordination structure of SACs via controlled structural evolution. As outlined in Section 4, such treatments directly influence key electronic descriptors, including coordination number, oxidation state, and d‐band center, by altering the bonding environment under oxidative, reductive, or inert atmospheres [143, 144, 145, 146]. For example, DeRita et al., investigated the in situ structural evolution of isolated Pt atoms in Pt_iso_/TiO_2_ catalysts under sequential pretreatments involving oxidation (300°C in O_2_), mild reduction (250°C in H_2_), and harsh reduction (450°C in H_2_), using Fourier transform infrared (FTIR) spectroscopy of CO adsorption at ‐140°C (Figure 6a) [147]. The vibrational frequencies of adsorbed CO shifted from 2,135 to 2,112 cm^−1^, and subsequently split into two peaks at 2077 and 2050 cm^−1^ after oxidation, mild reduction, and harsh reduction, respectively. These systematic red‐shifts reflect increased electron density at Pt centers and a corresponding shift in the d‐band center toward the Fermi level, leading to stronger CO adsorption. The above observations were further corroborated by in situ XAS analysis, which confirmed changes in the Pt‐O coordination number under different conditions. By comparing experimental and computational data, the authors proposed that oxidation promoted Pt incorporation into Ti_6c_ sites; mild reduction extracted Pt_iso_ from the lattice to form (PtO_2_)_ads_ species; and harsh reduction generated mobile (PtOH)_ads_ species capable of binding to both step and terrace sites (Figure 6b). In a later study, Lee et al. demonstrated that varying H_2_‐reduction temperatures can systematically tune the coordination number and oxidation state of Pt_1_ atoms in Pt_1_/CeO_2_‐Al_2_O_3_ [148]. The Pt L_3_‐edge Fourier transformed‐EXAFS (FT‐EXAFS) in Figure 6c showed a decrease in Pt‐O coordination from 5.9 in the calcined sample (Pt Cal) to 2.4 after H_2_ reduction at 500°C (Pt 500), while Pt 600 exhibited a Pt‐Pt coordination number of 7.9, indicating nanoparticle formation. Reduction at 300°C–500°C yielded coordinatively unsaturated Pt single atoms with lower Pt‐O coordination, as confirmed by XPS, which revealed a shift from highly oxidized Pt (Pt^0^: 16.6 at %) to predominantly metallic Pt (Pt^0^: 83.8 at %), indicating significant charge redistribution (Figure 6d). In CO oxidation, catalytic activity increased up to Pt 300, then slightly decreased for Pt 400 and Pt 500 (Figure 6e), reflecting a balance between adsorption strength and desorption kinetics governed by the Pt oxidation state and coordination environment.

**FIGURE 6:**
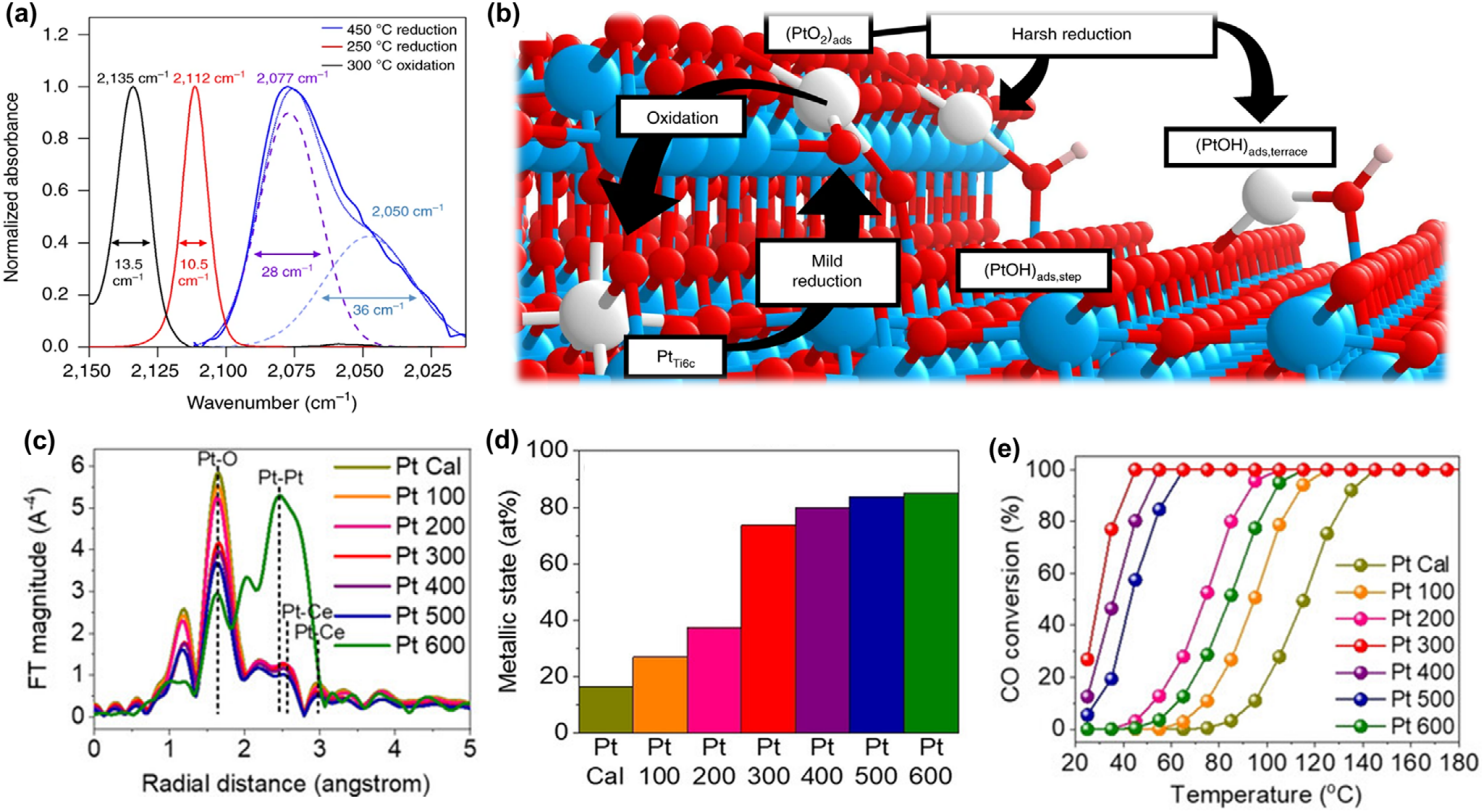
(a) FTIR spectra of CO adsorption at ‐140°C on Pt_iso_/TiO_2_ after sequential pretreatments via in situ oxidation (300°C in 760 torr O_2_), mild reduction (250°C in 760 torr H_2_) and harsh reduction (450°C in 760 torr H_2_). (b) Schematic showing the proposed dynamic evolution of Pt_iso_/TiO_2_ catalysts following oxidation, mild reduction, and harsh reduction. Reproduced with permission from ref [147]. Copyright 2019, Springer Nature. (c) Pt L_3_‐edge Fourier‐transformed‐EXAFS spectra of Pt_1_/CeO_2_‐Al_2_O_3_ reduced at various temperatures. (d) Atomic percentage of metallic Pt^0^ in Pt_1_/CeO_2_‐Al_2_O_3_, estimated from Pt 4d XPS spectra after thermal reduction at various temperatures. (e) CO oxidation activity of Pt SACs reduced at different temperatures (Pt‐Calc to Pt‐500), compared with conventional Pt nanoparticles (Pt‐600). Reproduced with permission from ref [148]. Copyright 2020, Wiley‐VCH.

Furthermore, high‐temperature treatments of carbon‐supported SACs have also been widely employed to tailor coordination structures and associated electronic properties. For instance, Wu and coworkers demonstrated a precise modulation of coordination numbers for atomically dispersed Co species anchored on nitrogen‐doped carbon (NC) through pyrolysis of a bimetallic Co/Zn zeolitic imidazolate framework (ZIF) at varying temperatures (800°C, 900°C, and 1000°C) [149]. During the pyrolysis process, Zn ions, owing to their low boiling point, evaporated, while Co ions were reduced by carbonized organic linkers. This process resulted in atomically dispersed Co atoms stabilized within the nitrogen‐doped porous carbon matrix, as clearly visualized in the HAADF‐STEM image. The FT‐EXAFS spectra revealed that the Co‐N coordination number progressively decreased from 4.1 to 2.2 when the pyrolysis temperature increased from 800°C to 1000°C (Figure 7a), accompanied by reduced nitrogen content. This decrease in coordination number resulted in an upshift of the Co d‐band center (from ‐1.06 eV for Co‐N_4_ to ‐0.81 eV for Co‐N_2_), enhancing the adsorption strength of key intermediates (Figure 7b). In electrochemical CO_2_ reduction, the Co‐N_2_ configuration exhibited the highest current density and achieved a CO Faradaic efficiency of approximately 95% at ‐0.68 V, significantly outperforming Co‐N_3_ and Co‐N_4_ counterparts (Figure 7c). In addition, Co‐N_2_ demonstrated excellent durability, maintaining stable current density and nearly constant CO Faradaic efficiency over 60 h of continuous operation. Density functional theory (DFT) calculations attributed this superior performance to the higher d‐band center of Co‐N_2_ (‐0.81 eV) compared with Co‐N_4_ (‐1.06 eV), resulting in stronger binding of the CO_2_* intermediate and thus enhanced catalytic activity. In another representative work, Lu and coworkers achieved coordination environment tuning of Pt single atoms through ALD onto pre‐engineered nitrogen‐doped carbon supports (Pt_1_/NC) containing controlled pyridinic nitrogen (N_pyri_) anchoring sites (Figure 7d) [150]. The NC supports were prepared by calcining precursors at different temperatures (from 600°C to 1000°C), systematically varying the abundance of N_pyri_ sites, followed by precise Pt atom deposition using ALD. FT‐EXAFS analysis showed that the Pt‐C/N coordination number increased from 3.0 for Pt_1_/NC‐600 to 3.8 for Pt_1_/NC‐1000‐3 h, accompanied by a substantial decrease in N_pyri_ content at higher calcination temperatures. This coordination evolution modulates the electronic structure of Pt, particularly by weakening Pt‐N_pyri_ interactions and upshifting the Pt 5d‐band center. Catalytic evaluation in the hydrogenation of para‐chloronitrobenzene (p‐CNB) revealed that four‐coordinated Pt_1_ SACs with reduced N_pyri_ content displayed remarkably high activity and outstanding recyclability, without detectable Pt leaching, aggregation, or structural degradation, outperforming lower‐coordination‐number counterparts (Figure 7e,f). Theoretical calculations further revealed that these higher‐coordination Pt sites possess lower formation energies (∼1 eV lower than three‐coordinated sites), contributing to improved stability against aggregation while maintaining favorable adsorption energetics for hydrogenation reactions.

**FIGURE 7:**
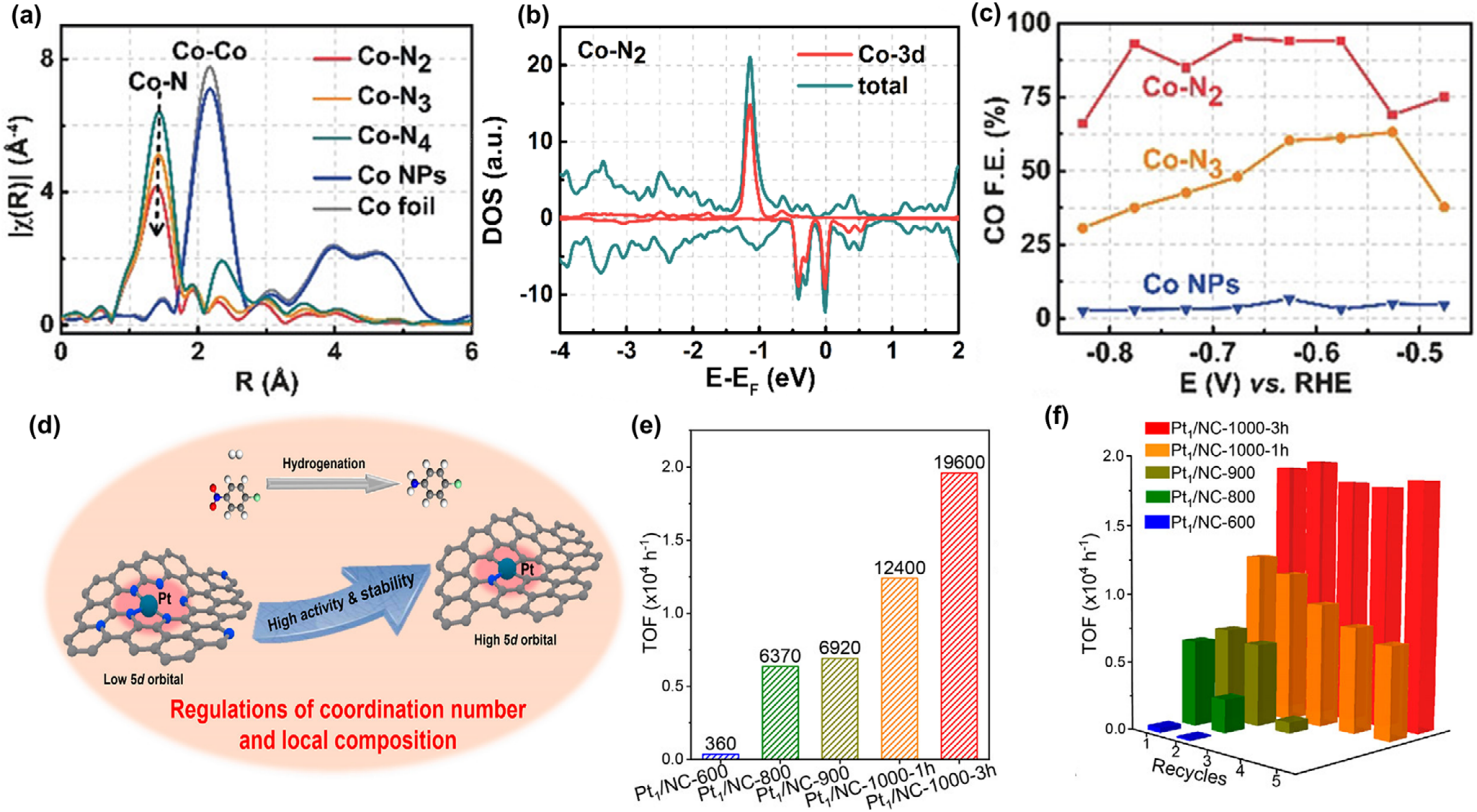
(a) FT‐EXAFS spectra at the Co K‐edge for Co‐N_2_, Co‐N_3_, and Co‐N_4_ SACs, illustrating variations in local coordination environments. (b) Total and partial density of states for Co‐N_2_. (c) CO Faradaic efficiencies at various applied potentials for different catalysts. Reproduced with permission from ref [149]. Copyright 2018, Wiley‐VCH. (d) Schematic representation of Pt_1_/NC SACs with tailored coordination number and local heteroatomic environment. (e) Catalytic activity and (f) recycling test of various Pt_1_/NC SACs in the hydrogenation of *para*‐chloronitrobenzene (p‐CNB). Reproduced with permission from ref [150]. Copyright 2021, American Chemical Society.

Overall, these studies highlight that thermal evolution provides a versatile route to dynamically tune coordination number, oxidation state, and electronic structure in SACs. By directly influencing key descriptors such as the d‐band center and charge distribution, thermal treatments enable precise control over adsorption energetics and catalytic pathways, thereby optimizing both activity and stability.

### 5.3 Dual‐Atom Pairing

Dual single‐atom catalysts (DSACs), also termed as single‐atom dimers (SADs), have recently emerged as a prominent research focus owing to their ability to achieve higher metal loadings and offer greater flexibility in tuning catalytic sites compared to conventional SACs [59, 151, 152, 153]. In these systems, when the distance between two catalytically active metal atoms is reduced to the sub‐nanometer scale, the overlap of their d–d orbitals facilitates the formation of hybrid orbitals, allowing heterometallic atomic pairs to act as a single cooperative active center [154]. This orbital coupling directly modifies key electronic descriptors such as the d‐band center, charge distribution, and orbital occupancy, thereby influencing adsorption energetics and reaction kinetics. Importantly, precise control over the interatomic spacing of these isolated metal sites provides a powerful means to tune catalytic performance [155]. For example, Lee and coworkers developed a straightforward strategy to synthesize Ni‐Co bimetallic SADs anchored on N‐doped carbon (NiCo‐SAD‐NC) by in situ trapping of metal ions followed by carefully controlled pyrolysis of N groups [59]. First‐principles calculations confirmed that the synergistic interaction between Ni and Co atoms at the atomic interface induces an upshift in the overall d‐band center, thereby accelerating water dissociation and optimizing proton adsorption, resulting in significantly enhanced HER kinetics under both alkaline and acidic conditions (Figure 8a,b). Aberration‐corrected HAADF‐STEM imaging and FT‐EXAFS analysis verified the successful formation of Ni‐Co bonds stabilized by six nitrogen atoms, with an average interatomic distance of 0.241 ± 0.024 nm, as determined by statistical analysis of multiple dimers (Figure 8c). Accordingly, the NiCo‐SAD‐NC demonstrated superior pH‐universal HER performance compared with NiCo nanoparticles and even rivaled commercial Pt/C. In another study, Han and coworkers explored the design of hydrogen oxidation reaction (HOR) catalysts through DFT screening and experimental validation [156]. Among various SACs and DSACs evaluated, Ru‐Ni DSACs were identified as the most promising active pairs. Structural characterization confirmed the formation of Ru‐Ni metallic bonds, while in situ XAS and DFT analyses revealed pronounced charge redistribution between Ru and Ni sites. This electronic interaction optimizes the adsorption energies of both H* and OH* intermediates, enabling a bifunctional mechanism in which Ru facilitates H_2_ dissociation while Ni promotes OH* adsorption. Such cooperative modulation of adsorption descriptors significantly enhances HOR kinetics.

**FIGURE 8:**
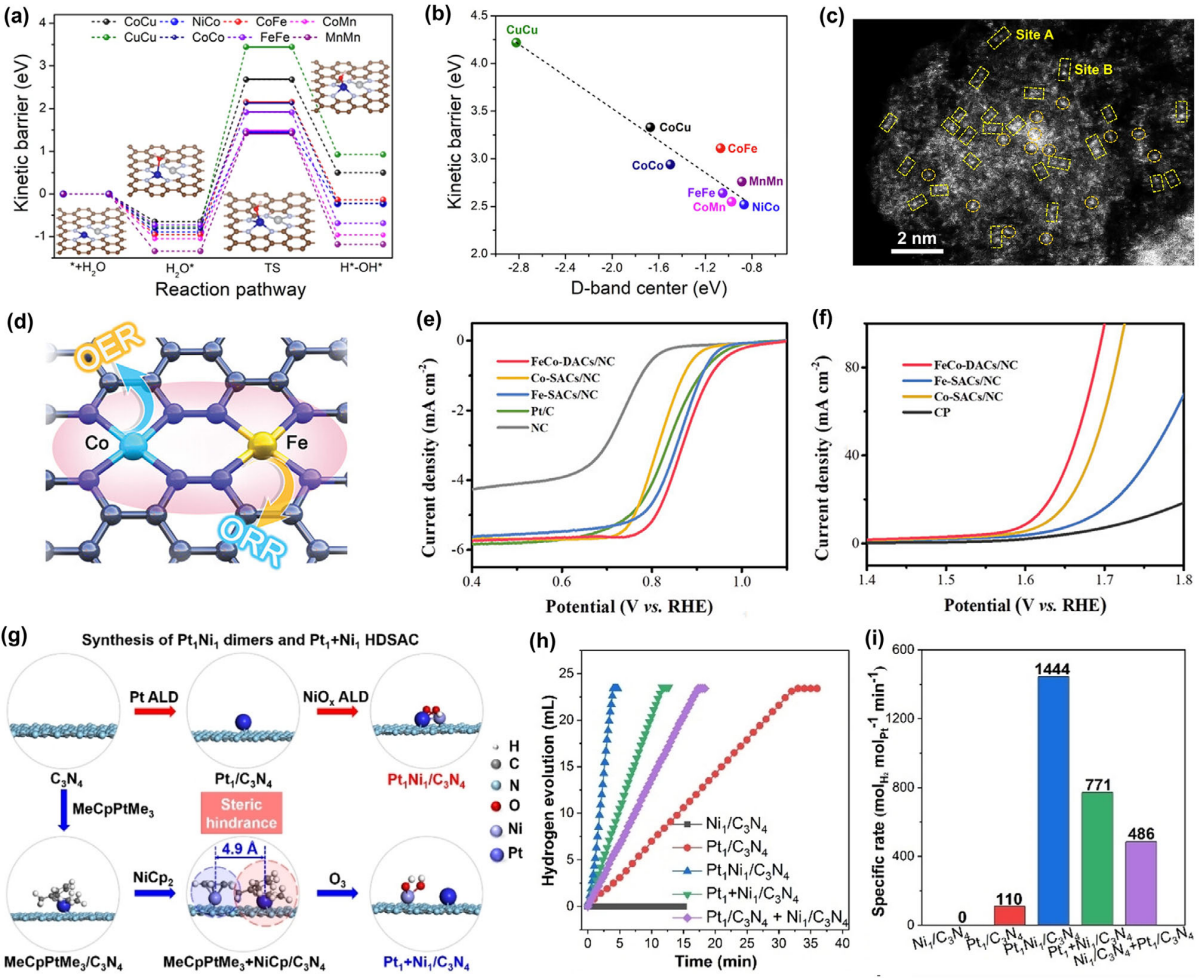
(a) Minimum energy paths for water dissociation reactions on TM‐SAD‐N_6_C. (b) Linear correlation between the d‐band center and kinetic energy barrier. (c) Aberration‐corrected HAADF‐STEM image of NiCo‐SAD‐NC, where yellow squares indicate dimer sites and orange circles highlight selected single Ni/Co atom sites. Reproduced with permission from ref [59]. Copyright 2021, Springer Nature. (d) Schematic representation of ORR and OER catalyzed by an Fe‐Co dual atom pair. LSV curves for the (e) ORR and (f) OER. Reproduced with permission from ref [157]. Copyright 2022, Wiley‐VCH. (g) schematic illustration of the synthesis of Pt_1_Ni_1_ oxo dimers and Pt_1_+Ni_1_ HDSAC. (h) Volume of hydrogen evolution versus reaction time for ammonia borane hydrolysis catalyzed by Ni_1_/C_3_N_4_, Pt_1_/C_3_N_4_, Pt_1_Ni_1_/C_3_N_4_, Pt_1_ + Ni_1/_C_3_N_4_ and the physical mixture of Ni_1_/C_3_N_4_ + Pt_1_/C_3_N_4_ at 25°C. (i) Specific rates of the above samples in panel (h) normalized to Pt loadings. Reproduced with permission from ref [158]. Copyright 2022, Wiley‐VCH.

Beyond enhancing single reactions, DSACs can also function as bifunctional catalysts by leveraging the complementary activities of each metal center. Bu and coworkers, for instance, synthesized contiguous FeN_4_ and CoN_4_ dual‐atom catalysts (DACs) uniformly embedded within N‐doped graphitic carbon (FeCo‐DACs/NC) [157]. These adjacent Fe and Co sites interact to narrow the band gap near the Fermi level, thereby facilitating electron transfer, while simultaneously serving as distinct catalytic centers for ORR and OER, respectively (Figure 8d). As a result, the FeCo‐DACs/NC exhibited outstanding oxygen electrocatalytic activity and superior rechargeable Zn‐air battery performance compared with their FeN_4_ and CoN_4_ counterparts (Figure 8e,f). Recently, Lu and colleagues devised an effective approach to regulate the interatomic distance between Pt and Ni atoms on g‐C_3_N_4_, enabling the synthesis of both Pt_1_Ni_1_ dimers and Pt_1_ + Ni_1_ heteronuclear dual‐single‐atom catalysts (HDSACs) by tuning steric hindrance during precursor assembly (Figure 8g) [158]. Spectroscopic studies revealed substantial electron transfer between Pt and Ni in the closely spaced Pt_1_Ni_1_ dimers, leading to significant modulation of the local electronic structure. In the case of ammonia borane dehydrogenation, the hydrogen formation rate was found to be inversely proportional to the Pt‐Ni interatomic distance. The Pt_1_Ni_1_/C_3_N_4_ catalyst with the closest Pt‐Ni spacing exhibited the highest H_2_ formation rate, approximately 13‐fold higher than that of the Pt_1_Ni_1_/C_3_N_4_ catalyst, highlighting the remarkable synergistic promotion between Pt and Ni (Figure 8h,i). This enhancement is attributed to optimized adsorption/desorption energetics arising from strong electronic coupling between Pt and Ni, highlighting the critical role of inter‐site electronic interactions in governing catalytic performance.

### 5.4 Heteroatom Doping

Heteroatom doping has emerged as a powerful strategy to engineer the local coordination environment of SACs, providing anchoring sites for stabilizing isolated metal atoms and modulating their electronic structure [159, 160, 161, 162]. By incorporating nonmetal heteroatoms such as N, S, P, B, or O into the carbon framework or oxide lattice, one can directly alter the local coordination geometry or act as an electronic promoter to fine‐tune the electron density, spin state, and oxidation state of the central metal atom. These electronic perturbations directly influence the adsorption energies of key intermediates, thereby governing catalytic activity and selectivity. For instance, N‐doped carbon frameworks are widely used to stabilize transition metals in M‐N_4_ moieties, while co‐doping with less electronegative heteroatoms (S/P) introduces asymmetry in the coordination sphere, leading to charge redistribution and spin‐state modulation that enhances catalytic reactivity [65]. For instance, Liu and coworkers employed a NaCl‐template pyrolysis method to prepare Fe SACs with Fe‐N/S bridging sites (Fe SA‐NSC‐900; contains FeN_3_S moieties) for ORR (Figure 9a) [163]. The FT‐EXAFS analysis confirmed coordination numbers of 2.8 for Fe‐N and 0.8 for Fe‐S, indicating the formation of an asymmetric FeN_3_S coordination environment. Compared with the symmetric Fe‐N_4_ configuration, the introduction of S induces charge redistribution and modifies the Fe 3d electronic structure, resulting in a moderate upshift of the d‐band center (higher density of states near the Fermi level) and alteration of the spin configurations (N (2s^2^2p^3^) has fewer valence electrons than S (3s^2^3p^4^), replacing N with S will lead to more occupied valence orbitals). This leads to optimized adsorption of oxygenated intermediates by weakening overly strong binding, thereby accelerating ORR kinetics. Consequently, the Fe SA‐NSC‐900 catalyst exhibited superior ORR activity compared to Fe‐N_4_ analogues and even Pt/C (Figure 9b). Similarly, Liang and coworkers employed a sulfur‐tethering strategy to stabilize noble metal SACs at high loading, with sulfur dopants serving as strong metal‐S anchors within mesoporous carbon, enabling high dispersion and improved durability [164].

**FIGURE 9:**
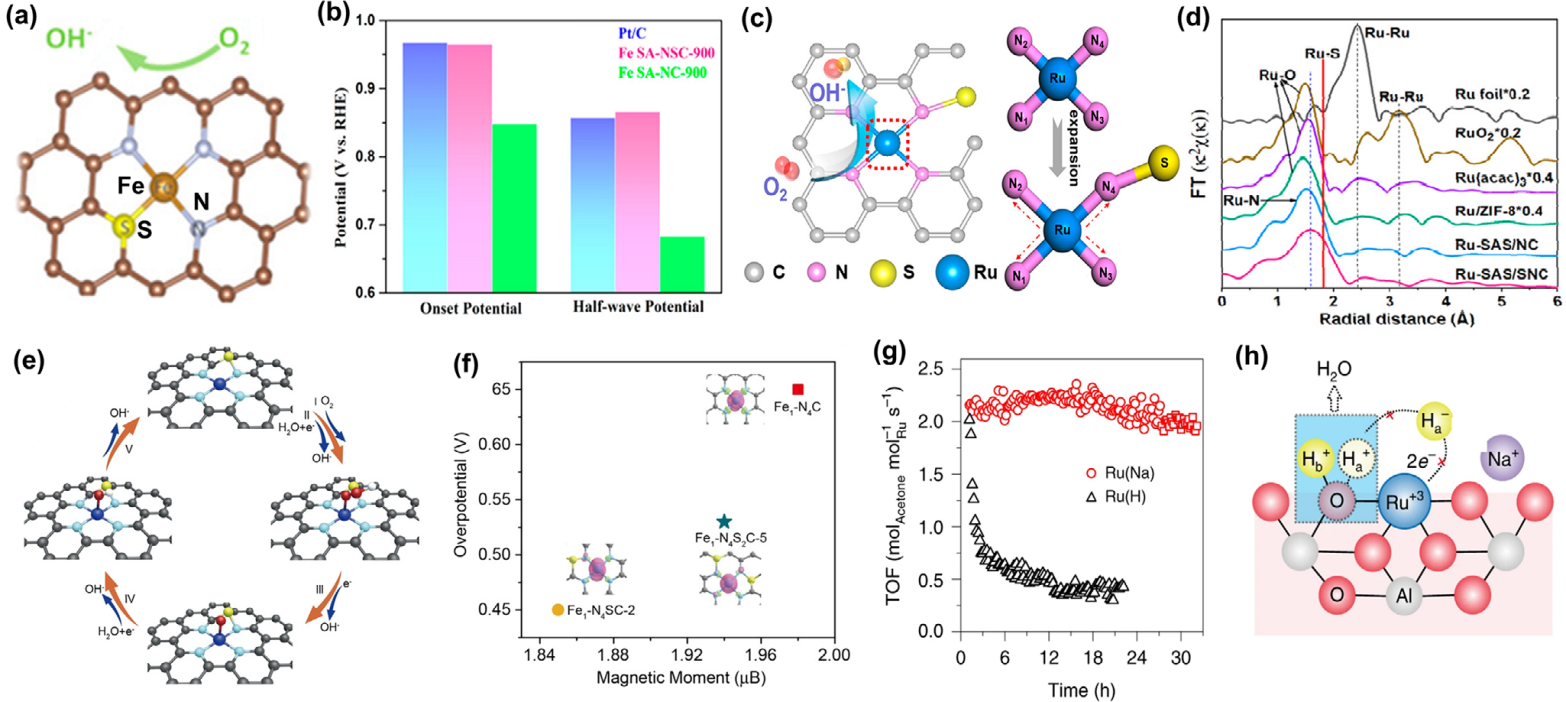
(a) Schematic illustration of Fe SA‐NSC‐900 catalyst for ORR and (b) onset potential and half‐wave potential for Fe SA‐NSC‐900, Fe SA‐NC‐900, and Pt/C. Reproduced with permission from ref [163]. Copyright 2021, American Chemical Society. (c) Schematic atomic models of RuN_4_‐S and RuN_4_. (d) Ru K‐edge FT‐EXAFS signal of Ru‐SAS/SNC, along with reference samples. Reproduced with permission from ref [165]. Copyright 2022, American Chemical Society. (e) The proposed ORR mechanism for the Fe_1_‐N_4_SC. (f) ORR overpotential as a function of magnetic moments. Reproduced with permission from ref [162]. Copyright 2021, Wiley‐VCH. (g) Acetone hydrogenation catalyzed by Ru(Na) and Ru(H) at 150°C with 3 MPa H_2_ and (h) schematic diagram of the function of Na^+^ in securing the hydride. Reproduced with permission from ref [166]. Copyright 2020, Springer Nature.

Beyond first‐shell coordination tuning, heteroatom substitution in the second coordination shell offers an additional avenue to modulate the electronic structure while preserving the stability of M‐N_4_ motifs. For example, Qin et al. introduced a sulfur‐anion coordination strategy in Ru‐N‐C catalysts (Ru‐SAS/SNC), where S bonded to N in the second coordination sphere, effectively tuning the electronic configuration of Ru, while maintaining the Ru‐N_4_ core (Figure 9c) [165]. FT‐EXAFS spectra confirmed that Ru‐SAS/SNC exhibited features more consistent with Ru‐N bonding than Ru‐S, validating the preservation of Ru‐N_4_ coordination (Figure 9d). The RuN_4_‐S units in Ru‐SAS/SNC present a lower d‐band center in comparison with Ru‐SAS/NC (‐0.95 over ‐0.15 eV), where the decrease in intermediates’ adsorption free energy contributes to the increased ORR activity of Ru‐SAS/SNC, revealed by theoretical calculations. This subtle modulation of the coordination environment of Ru‐SAS/SNC resulted in superior ORR performance (activity and durability) compared with conventional Ru‐SAS/NC catalysts and even commercial Pt/C. In another representative study, sulfur doping was shown to modulate the spin state of Fe centers in Fe‐N‐C SACs by introducing S atoms into the second coordination sphere, forming a C‐FeN_4_‐S configuration (Figure 9e,f) [162]. Spectroscopic analyses, including ^57^Fe Mössbauer and EPR, revealed that S incorporation induces a transition to a low‐spin Fe^3+^ state, which was identified operando as the active site for ORR. From an electronic perspective, this ligand‐field modification alters Fe 3d orbital occupancy and effectively tunes the d‐band characteristics. DFT calculations further showed that the low‐spin state weakens OH* adsorption, facilitating its desorption and thereby enhancing ORR kinetics. As a result, the S‐doped catalyst exhibited superior activity compared to conventional Fe‐N‐C systems, highlighting spin‐state engineering as an effective descriptor for SAC optimization.

In addition to non‐metal heteroatoms, alkali ions have also been exploited as electronic promoters to regulate SAC activity. Zheng et al. demonstrated that Na incorporation into Ru/Al_2_O_3_ significantly improved the hydrogenation of acetone to isopropanol (Figure 9g) [166]. The presence of Na^+^ ions induces electronic polarization around Ru sites, modifying their electron density and stabilizing key hydride intermediates. This effect can be interpreted as a shift in the effective electronic structure of Ru, influencing adsorption energetics and reaction pathways. The Na‐modified catalyst not only showed higher activity but also exhibited enhanced durability compared to un‐doped Ru/Al_2_O_3_, which underwent rapid deactivation under the same conditions. Mechanistic studies revealed that Na^+^ ions stabilized hydride species by preventing their migration to interfacial oxygen, thereby suppressing Ru reduction and aggregation and ensuring the long‐term stability of isolated Ru sites (Figure 9h). Collectively, these studies highlight heteroatom doping as a versatile and effective strategy for tuning key electronic descriptors including d‐band center, charge distribution, and spin state, thereby enabling precise control over catalytic activity, selectivity, and stability. Extending such approaches to diverse support systems and heteroatom combinations offers significant opportunities for the rational design of next‐generation SACs for a wide range of electrochemical and thermocatalytic processes.

### 5.5 Single‐Atom/Nanocluster or Nanoparticle Interfacial Synergy

In supported metal catalysts, different metallic species such as isolated single atoms, nanoclusters, and nanoparticles often coexist on the same support surface. The interfacial interactions among these distinct entities, particularly between single atoms and neighboring clusters or nanoparticles, can generate unique synergistic effects that substantially modify the overall catalytic behavior [167]. Unlike conventional SACs, where the activity is primarily governed by the local coordination environment of the single atom, coupling with adjacent metal ensembles introduces new coordination motifs and electronic interactions. These interactions can induce charge redistribution, modify the d‐band center, alter spin states, and tune the oxidation state of the single atoms, thereby optimizing adsorption energetics and stabilizing reactive intermediates [168]. A representative example of this strategy was reported by Peng and coworkers, who demonstrated an effective approach to optimize the ORR activity of Fe single atoms by incorporating Fe clusters (Fe‐ACSA@NC) through a coating‐pyrolysis‐etching route [169]. HAADF‐STEM image confirmed the coexistence of Fe single atoms and Fe clusters in Fe‐ACSA@NC (Figure 10a). Furthermore, the Fe K‐edge FT‐EXAFS spectra revealed the presence of both Fe‐N and Fe‐Fe bonds, characteristic of Fe single atoms and clusters, respectively (Figure 10b). The as‐prepared Fe‐ACSA@NC displayed excellent ORR activity, outperforming Fe‐SA@NC, Fe‐NPsSA@NC, and commercial Pt/C. Mechanistic studies indicated that Fe clusters induce charge transfer to adjacent Fe‐N_x_ sites, leading to a redistribution of electron density and a modulation of the Fe 3d electronic structure. This electronic modulation weakened the OH* binding strength, facilitated desorption, and enabled superior 4e^−^ ORR activity, making Fe‐ACSA@NC a highly efficient cathode catalyst for metal‐air batteries (Figure 10c). In a parallel study, Hou and coworkers reported the development of an ultra‐low Ru catalyst by integrating atomically layered Ru nanoclusters with adjacent Ru single atoms [170]. This unique configuration employed a bridging‐Ru‐H activation strategy to accelerate HER kinetics (Figure 10d). The resulting hybrid Ru catalyst demonstrated excellent HER activity in alkaline media, requiring an exceptionally low overpotential and outperforming benchmark Ru/C and Pt/C catalysts. DFT calculations revealed that charge transfer between Ru single atoms and nanoclusters modifies the local electronic structure, including the d‐band center energy of Ru SAs, facilitating optimal adsorption of H* intermediates. The formation of a bridged Ru‐H configuration between single atoms and clusters promotes proton transfer and accelerates Volmer–Heyrovsky steps (Figure 10e). Beyond homometallic systems, heterometallic coupling of single atoms with nanoclusters has also been shown to profoundly influence catalytic properties. For instance, Chu et al., demonstrated that the integration of Pt single atoms with Ru clusters significantly enhanced HER performance [171]. In this case, Pt single atoms effectively reduced the water dissociation energy barrier by modulating the electronic structure of Ru, thereby amplifying the intrinsic activity of Ru clusters.

**FIGURE 10:**
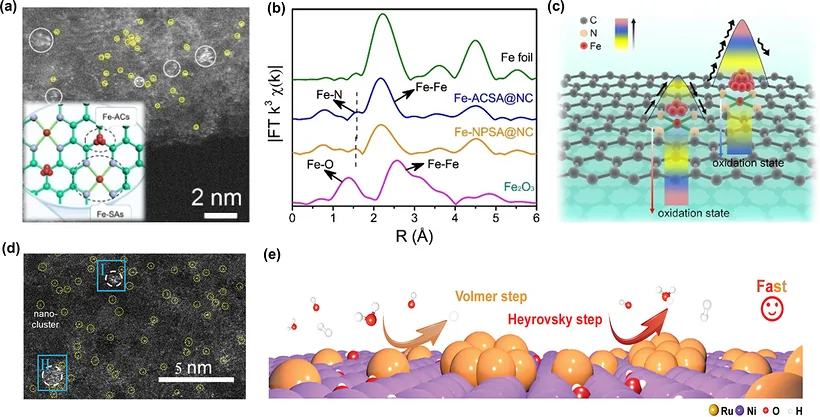
(a) Aberration‐corrected STEM image of Fe‐ACSA@NC. (b) Fourier‐transformed Fe K‐edge EXAFS spectra of Fe‐ACSA@NC compared with corresponding control samples. (c) Illustration of the correlation between ORR activity and the oxidation state of Fe single‐atom sites in the presence of Fe clusters. Reproduced with permission from ref [169]. Copyright 2022, Wiley‐VCH. (d) HAADF‐STEM image of NS‐Ru@NiHO/Ni_5_P_4_. (e) Proposed schematic representation of the HER mechanism involving both Ru single atoms and Ru clusters. Reproduced with permission from ref [170]. Copyright 2023, Wiley‐VCH.

In addition to nanoclusters, the incorporation of nanoparticles with SACs provides another strategy to achieve synergistic catalysis by tailoring the electronic structure of the single atoms, including their charge distribution, d‐band center, and spin state. Zheng and coworkers designed a hybrid catalyst consisting of Pd single atoms and Pd nanoparticles anchored on graphitic carbon nitride (Pd_1+NPs_/C_3_N_4_) for photocatalytic CO_2_ reduction to CH_4_ (Figure 11a,b) [172]. The Pd_1+NPs_/C_3_N_4_ catalyst achieved a remarkable 97.8% selectivity for CH_4_ production along with high yield rates in pure water. Mechanistic investigations revealed that Pd single atoms were primarily responsible for CO_2_ activation, whereas Pd nanoparticles facilitated water dissociation to enhance H* availability through hydrogen spillover. This cooperative mechanism promoted CO_2_ hydrogenation at Pd single‐atom sites, resulting in enhanced CH_4_ production (Figure 11c). A similar concept of species heterogeneity was reported by Ma and coworkers, who explored the cooperative effect between isolated Rh single atoms and Rh clusters/nanoparticles in cyclohexanol dehydrogenation [173]. They found that Rh single atoms (Rh_1_/ND@G) were highly active for the initial dehydrogenation step from cyclohexanol to cyclohexanone, achieving 98% selectivity, whereas Rh clusters and nanoparticles (Rh_n_ and Rh_p_) were more effective in catalyzing the subsequent transformation from cyclohexanone to phenol (Figure 11d). From an electronic perspective, the distinct coordination environments of Rh_1_ and Rh_n_/Rh_p_ lead to different electronic structures and adsorption preferences for reaction intermediates. The mixed catalyst containing both Rh single atoms and Rh clusters/nanoparticles (Rh_1+n_/NG@G) exhibited the highest H_2_ formation rate with phenol as the main product, highlighting the critical role of structural heterogeneity in driving multistep reactions (Figure 11e).

**FIGURE 11:**
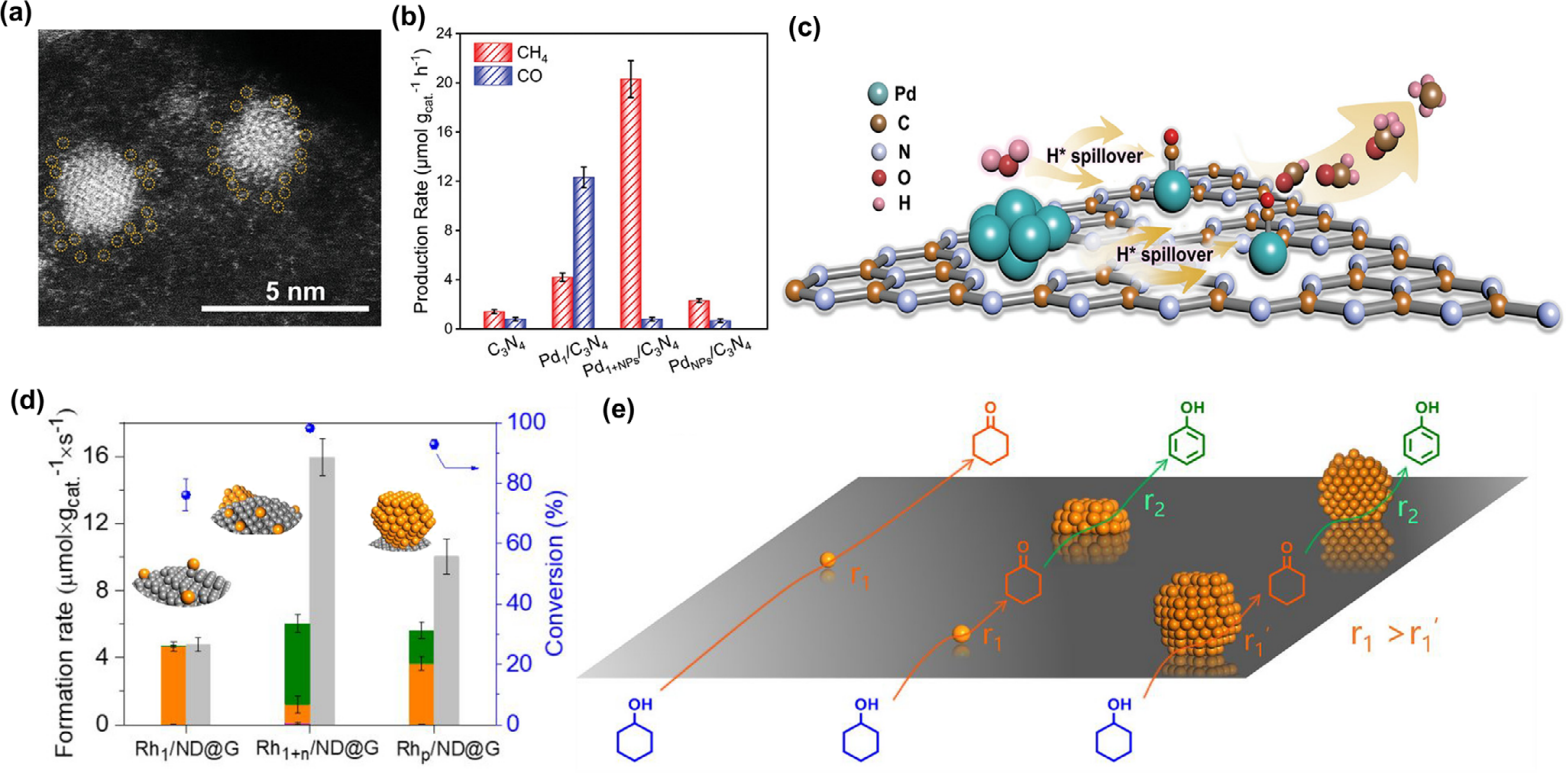
(a) Aberration‐corrected HAADF‐STEM image of Pd_1_+NPs/C_3_N_4_. (b) Comparison of CH_4_ and CO production rates during CO_2_ photoreduction over C_3_N_4_, Pd_1_/C_3_N_4_, Pd_1_+NPs/C_3_N_4_, and Pd NPs/C_3_N_4_. (c) Proposed reaction mechanism for the photocatalytic conversion of CO_2_ to CH_4_ on the synergetic Pd catalyst. Reproduced with permission from ref [172]. Copyright 2022, Wiley‐VCH. (d) Catalytic performance of cyclohexanol dehydrogenation over Rh_1_/ND@G, Rh_1+n_/ND@G, and Rh_p_/ND@G catalysts. (e) Schematic illustration of hydrogen generation pathways from cyclohexanol on Rh_1_/ND@G, Rh_1+n_/ND@G, and Rh_p_/ND@G. Reproduced with permission from ref [173]. Copyright 2022, American Chemical Society.

## 6 Impact of SACs Coordination Modulation on Electrocatalysis

In Sections 4 and 5, we established that modulation of the local coordination environment in SACs directly governs key electronic descriptors, including charge distribution, d‐band center position, orbital occupancy, and spin‐state, through rational design strategies. Building on this foundation, the present section outlines how such descriptor‐level changes translate into catalytic performance by regulating the adsorption energetics of reaction intermediates and the associated kinetic barriers in electrocatalytic processes such as the OER, HER, ORR, and CO_2_RR. In recent years, SAC‐based electrocatalysis has garnered significant attention, largely because the coordination environment surrounding the isolated metal center, including atoms in the first, second, and even higher coordination spheres, plays a decisive role in determining catalytic function [11, 19, 59, 81, 174, 175]. These coordination features not only introduce additional adsorption sites but also fine‐tune intermediate binding strengths and reaction pathways, thereby enabling highly selective and efficient catalytic behavior [176, 177]. The following subsections summarize recent advances in coordination‐engineered SACs across different electrocatalytic reactions, with particular emphasis on how specific coordination motifs regulate electronic structure and, consequently, control activity, selectivity, and stability at the atomic level.

### 6.1 Oxygen Evolution Reaction

The electrochemical OER constitutes the anodic half‐reaction in water splitting and is a key process in energy technologies, including water electrolyzers, fuel cells, and metal‐air batteries [178, 179]. Owing to the involvement of four proton‐electron transfer steps, the OER is both thermodynamically demanding and kinetically sluggish, requiring high overpotentials. In line with the descriptor‐based framework discussed in Sections 4 and 5, the OER activity of SACs is primarily governed by how the local coordination environment modulates the electronic structure of the active site, thereby tuning the adsorption energetics of key oxygenated intermediates. In acidic and alkaline media, the reaction proceeds through distinct elementary steps involving surface‐bound oxygen intermediates [180]:

In alkaline medium:

$$*+\;OH^{-}\;\to \;e^{-}+OH^{*}$$

$$OH^{-}+\;OH^{*}\;\to H_{2}O+e^{-}+O^{*}$$

$$OH^{-}+O^{*}\;\to e^{-}+\mathrm{OO}H^{*}$$

$$OH^{-}+\;\mathrm{OO}H^{*}\;\to H_{2}O+e^{-}+O_{2}+*$$

(*denotes active site)

In acidic medium:

$$H_{2}O\;\to \;H^{+}+OH^{*}+e^{-}$$

$$OH^{*}\;\to \;H^{+}+O^{*}+e^{-}$$

$$H_{2}O+O^{*}\;\to \;H^{+}+\mathrm{OO}H^{*}+e^{-}$$

$$\mathrm{OO}H^{*}\;\to \;H^{+}+*+e^{-}+O_{2}\;$$

To date, the adsorbate evolution mechanism (AEM) and the lattice‐oxygen‐mediated mechanism (LOM) have been reported as the underlying mechanisms of OER [19]. The OER proceeds through four sequential proton‐electron transfer steps involving the formation and conversion of HO*, O*, and HOO* intermediates. The catalyst must provide an energetically favorable surface where each step has a minimized transition‐state barrier, which is largely dictated by how the active site stabilizes these intermediates. In the conventional AEM, a linear scaling relationship between HO* and HOO* adsorption energies imposes a theoretical overpotential limit of ∼370 mV, restricting intrinsic activity. In contrast, the LOM bypasses the formation of HOO* by enabling direct O─O bond formation through the participation of lattice oxygen [181]. This pathway effectively breaks the adsorption‐energy scaling constraints and can thus afford lower theoretical overpotentials. In both mechanisms, the rate‐determining step corresponds to the elementary reaction step with the highest Gibbs free‐energy barrier, dictating the overall kinetics and catalytic performance [182]. Consequently, tailoring the coordination environment in SACs through strategies such as axial ligand incorporation, heteroatom doping, and metal‐support interaction engineering (Section 5), provides a rational route to modulate electronic descriptors (e.g., d‐band center, oxidation state, and orbital occupancy), thereby optimizing intermediate binding and enabling highly active OER electrocatalysts that can surpass conventional Ir/Ru‐based oxides.

Several recent studies highlight the importance of coordination engineering in enabling superior OER activity [183, 184]. For instance, Wang and coworkers developed penta‐coordinated Co single sites by introducing axial O ligands into a CoN_4_ framework supported on MXene nanosheets (CoN_4_‐O/MX) [185]. The Co K‐edge FT‐EXAFS analysis revealed well‐defined Co‐N and Co‐O coordination environments with coordination numbers of 4.0 and 1.0, respectively. The CoN_4_‐O/MX exhibited excellent alkaline OER activity, reaching 10 mA/cm^2^ at 1.55 V, surpassing the performance of other catalysts and highlighting the positive impact of axial O regulation on enhancing OER activity (Figure 12a). Notably, axial O coordination induces spin polarization in the Co 3d orbitals, where the spin‐down dz^2^ orbital shifts above the Fermi level, resulting in an unsaturated electronic configuration. This also reflects a redistribution of 3d electron occupancy and partial electron depletion at the Co center, consistent with enhanced charge delocalization (Figure 12b). This spin‐state modulation upshifts the d‐band center and enhances the Lewis basicity of Co sites, thereby facilitating charge transfer to OER intermediates and accelerating reaction kinetics. Similarly, Feng et al., demonstrated that co‐doping of S and N atoms into the carbon support of a Ni‐SAC (S|NiN_x_‐PC/EG) significantly enhances OER performance, achieving an overpotential of 280 mV at 10 mA/cm^2^ and a Tafel slope of 45 mV dec^−1^ [186]. Comparative analysis revealed that replacing one N ligand with S in the Ni‐N_4_ motif induces asymmetric charge redistribution and modifies the local electronic structure, effectively tuning the adsorption energies of HO*, O*, and HOO* intermediates. In another example, Chen and coworkers synthesized a S/N co‐doped carbon support hosting asymmetric Fe–Fe diatomic sites (*A*‐Fe_2_S_1_N_5_/SNC), which required only 193 mV to reach 10 mA cm^−2^, surpassing commercial RuO_2_ (Figure 12c) [187]. Operando XAFS measurements showed dynamic modulation of Fe valence and Fe‐N/S/Fe coordination shells during OER, indicating continuous evolution of electronic structure under working conditions. This dynamic coordination enables optimized adsorption configurations for O*, OH*, and OOH* species on adjacent Fe centers (Figure 12d,e). The proposed reaction route over the 3O‐Fe_2_S_1_N_5_/SNC catalyst is shown in Figure 12f.

**FIGURE 12:**
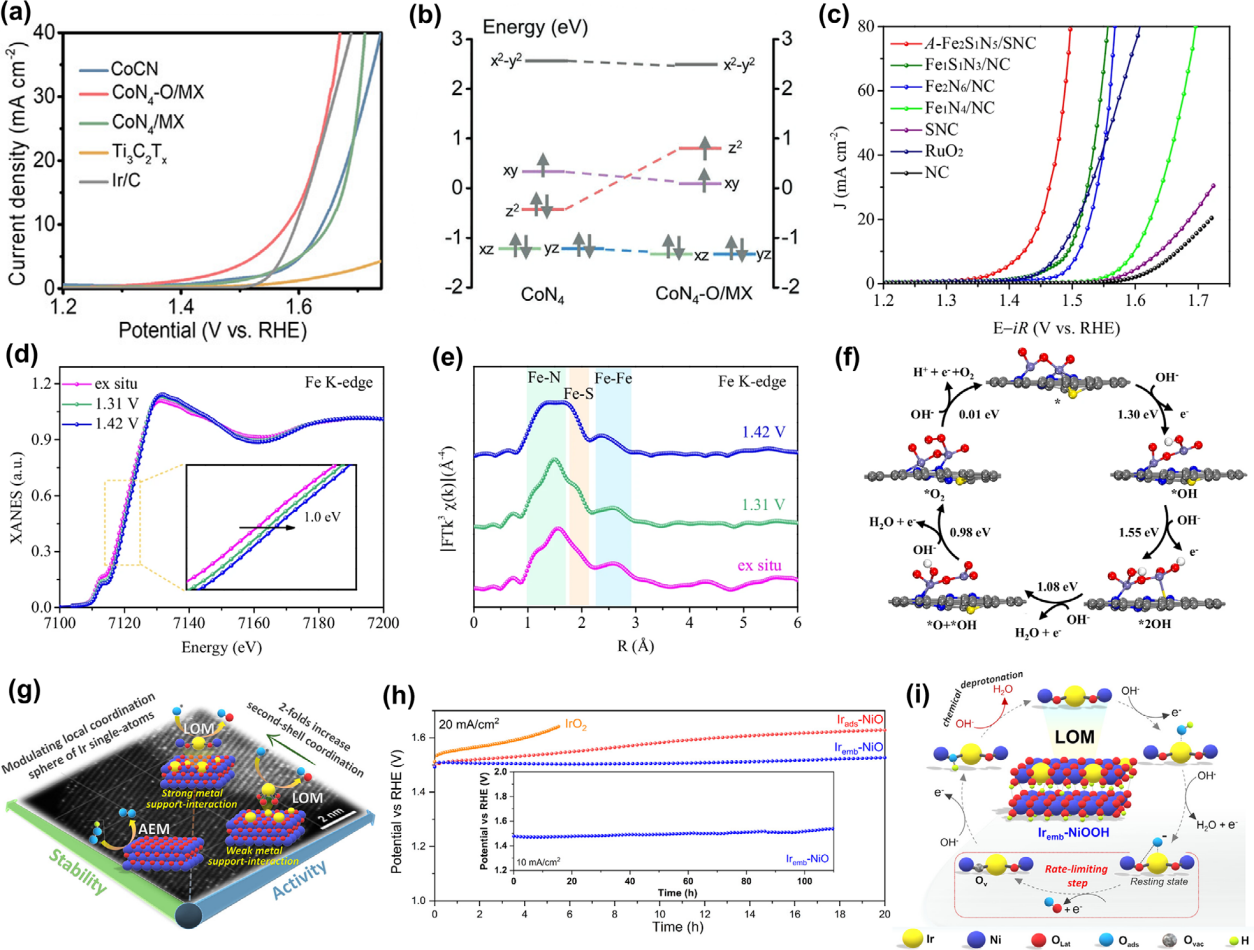
(a) The OER LSV polarization of CoN_4_‐O/MX with other control samples in 1 M KOH. (b) Co 3d orbital splitting of Co centers in CoN_4_‐O/MX and CoN_4_. Reproduced with permission from ref [185]. Copyright 2022, Wiley‐VCH. (c) The OER LSV polarization of *A*‐Fe_2_S_1_N_5_/SNC in 1 M KOH along with other comparison samples. The *operando* Fe K‐edge XANES (d) and FT‐EXAFS (e) spectra for *A*‐Fe_2_S_1_N_5_/SNC at ex situ, 1.31, and 1.42 V versus RHE. (f) The proposed mechanism of the OER on *A*‐Fe_2_S_1_N_5_/SNC. Reproduced with permission from ref [187]. Copyright 2024, Springer Nature. (g) Schematic illustration of second‐coordination sphere modulated Ir single atoms on NiO catalyzing OER via lattice oxygen mechanism (LOM) pathway. (h) The long‐term alkaline OER stability test for Ir_emb_‐NiO along with control samples. (i) The proposed schematic illustration of the LOM process for Ir_emb_‐NiO (reconstructed to Ir_emb_‐NiOOH). Reproduced with permission from ref [19]. Copyright 2024, American Chemical Society.

In our recent study, we demonstrated that second‐shell coordination modulation of Ir single atoms on NiO significantly enhances both mass activity and stability while maintaining an unconventional LOM pathway (Figure 12g) [19]. Ir atoms partially embedded within the NiO surface (Ir_emb_‐NiO) exhibited a two‐fold increase in Ir‐Ni second‐shell coordination relative to surface‐adsorbed Ir (Ir_ads_‐NiO), indicative of stronger metal‐support interactions. This structural modulation alters the electronic environment of Ir sites, promoting the formation of high‐valent Ir species and facilitating lattice oxygen participation. This catalyst achieved superior mass activity and remarkable long‐term durability (degradation rate: ∼1 mV/h), outperforming commercial IrO_2_ and conventional Ir_ads_‐NiO (Figure 12h). Operando XAS and Raman spectroscopy confirmed the formation of high‐valent Ir species on a NiOOH surface during OER, validating the involvement of the LOM and revealing a pathway that circumvents traditional scaling relationships (Figure 12i).

### 6.2 Hydrogen Evolution Reaction

The HER constitutes the cathodic half‐reaction in water splitting and proceeds through a two‐electron transfer process involving adsorbed hydrogen intermediates (H*) [4, 59]. As discussed in Section 4, the free energy of hydrogen adsorption (Δ*G*_H*_) serves as a primary descriptor for HER activity, as it reflects the binding strength between H* and the catalytically active sites. An ideal HER catalyst exhibits a Δ*G*_H*_ value close to zero, ensuring balanced adsorption and desorption of hydrogen intermediates [188]. Depending on the electrolyte environment, HER typically proceeds through either the Volmer–Tafel or Volmer–Heyrovsky mechanism (Figure 13) [3, 189]:

**FIGURE 13:**
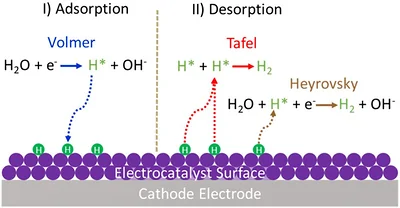
Schematic diagram of the Volmer, Tafel, and Heyrovsky processes on the surface of an electrocatalyst. Reproduced with permission from ref [3]. Copyright 2024, American Chemical Society.

In acidic medium:

$$e^{-}+H^{+}\to \;H^{*}\;\;\;\;\;\left(Volmer\;step\right)$$

$$e^{-}+H^{+}+H^{*}\to \;H_{2}\;\;\;\;\;\left(Heyrovsky\;step\right)$$

$$H^{*}+H^{*}\;\to \;H_{2}\;\;\;\;\;\left(Tafel\;step\right)$$

(*denotes active site)

In alkaline medium:

$$e^{-}+H_{2}O\;\to \;H^{*}+OH^{-}\;\;\;\left(Volmer\;step\right)$$

$$e^{-}+H_{2}O\;+H^{*}\;\to \;H_{2}+OH^{-}\;\left(Heyrovsky\;step\right)$$

$$H^{*}+H^{*}\;\to \;H_{2}\;\;\;\;\;\left(Tafel\;step\right)$$

The high energy barriers associated with these steps necessitate catalysts that effectively minimize activation energies. In this context, coordination modulation strategies outlined in Section 5 provide an effective route to tune Δ*G*_H*_, primarily by adjusting the d‐band center, charge distribution, and local electronic structure of SACs. Such control enables optimization of intrinsic kinetics and enhancement of turnover frequencies through maximized atom utilization [141]. For instance, Wang and coworkers synthesized a series of Ni‐SACs with systematically modulated first‐shell coordination environments through heteroatom co‐doping (B, P, S, N) and explored the effect of different coordination environments of Ni‐SACs on HER catalytic activity [190]. Among electrocatalysts, the Ni‐SAC with the Ni‐N_3_‐P structure (Ni‐N, P/CNFs) delivered the best acidic HER performance (needed 38 mV to reach 10 mA cm^−2^), attributed to a favorable *d*‐band center enabling optimal H* adsorption (Figure 14a). DFT calculations further confirmed that Ni‐N_3_P possesses the lowest Δ*G*_H*_, correlating with faster HER kinetics (Figure 14b). Axial coordination represents another important approach for tuning local electronic structure. Zhang et al., prepared Pt SACs on NiFe‐LDH through an irradiation‐impregnation method, incorporating different axial ligands (F, Cl, Br, I, OH), forming X‐Pt/LDH materials [191]. The FT‐EXAFS fitting results of Cl‐Pt/LDH revealed a tetrahedral geometry for the Pt site, arising from combined Pt‐O and Pt‐Cl coordination (Figure 14c). Among all the samples, Cl‐Pt/LDH showed the highest alkaline HER activity, suggesting that the HER activity of Pt‐SACs was significantly influenced by axial ligand coordination (Figure 14d). In‐depth mechanistic analysis indicated that the chloride ligand optimizes both OH* and H* adsorption due to its high electron affinity, thus promoting the Volmer step during water dissociation.

**FIGURE 14:**
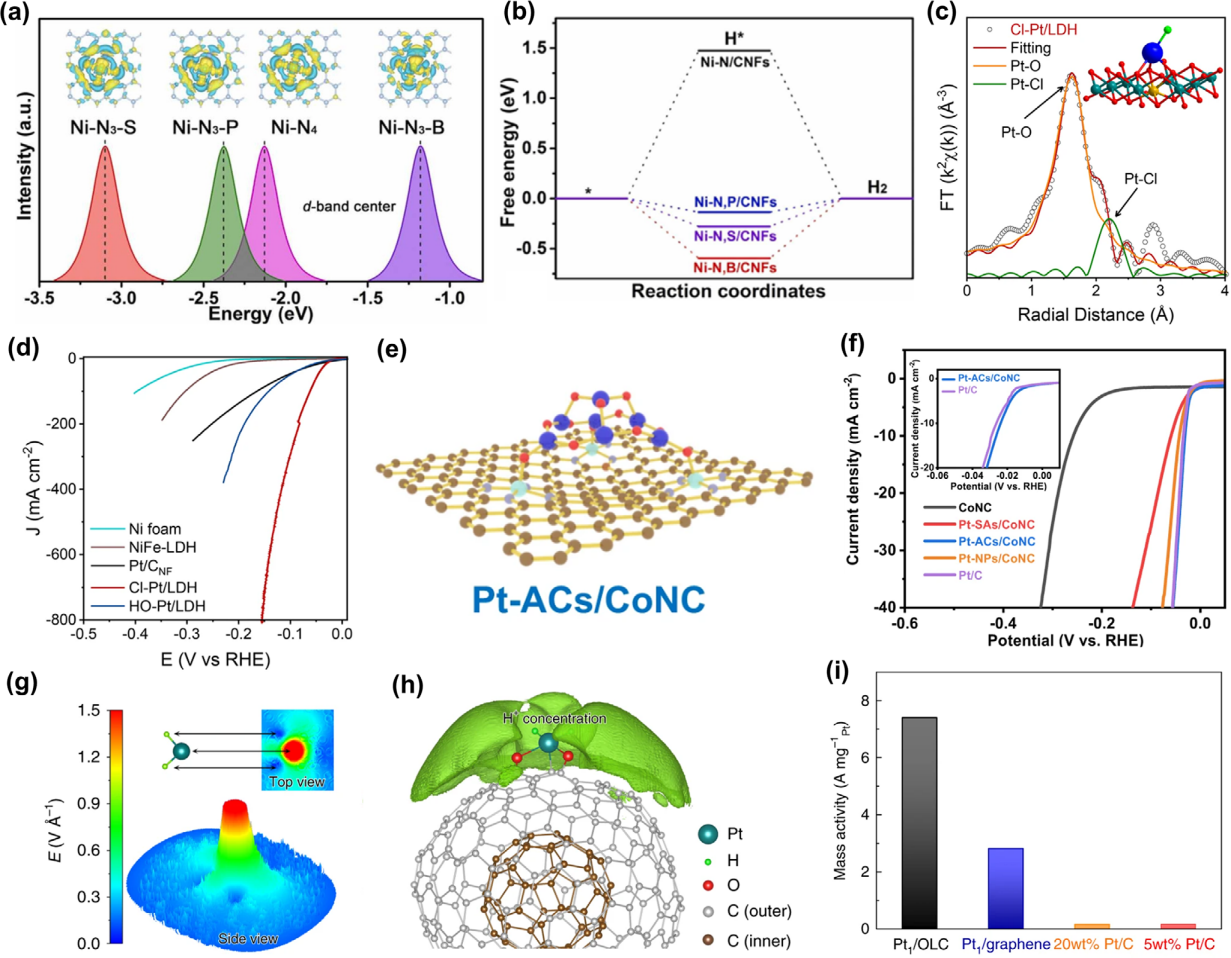
(a) The d‐Band centers of Ni single atoms in different local coordination environments. (b) The absorption Gibbs free energy of H* on Ni‐SACs with different coordination environment in the HER steps. Reproduced with permission from ref [190]. Copyright 2022, Elsevier. (c) The FT‐EXAFS fitting curve (inset is the magnified local structure) of Cl‐Pt/LDH (The blue, olive, yellow, red, green, and gray spheres refer to Pt, Ni, Fe, O, Cl, and H atoms, respectively). (d) The HER polarization curves of Ni foam, NiFe‐LDH, Pt/C, Cl‐Pt/LDH, and HO‐Pt/LDH in alkaline medium. Reproduced with permission from ref [191]. Copyright 2022, Springer Nature. (e) Schematic illustration of Pt‐ACs/CoNC. (f) The HER polarization curves of CoNC, Pt‐SAs/CoNC, Pt‐ACs/CoNC, Pt‐NPs/CoNC and Pt/C with the same total loading in acidic solution. Reproduced with permission from ref [192]. Copyright 2022, Springer Nature. (g) The electric field map and (h) model scheme of the H_2_Pt_1_/OLC system at the tip‐like Pt site with an equilibrium potential. (i) The HER mass activity of Pt_1_/OLC normalized to the Pt loading at an *η* of 38 mV with respect to the reference catalysts. Reproduced with permission from ref [142]. Copyright 2019, Springer Nature.

Beyond isolated single‐atom sites, interfacial synergy effects (Section 5.5) further enhance HER performance by introducing cooperative electronic interactions. Lu and coworkers engineered Pt atomic clusters containing Pt‐O‐Pt motifs anchored adjacent to isolated Co atoms on N‐doped carbon (Pt‐ACs/CoNC) (Figure 14e) [192]. The Pt‐ACs/CoNC displayed excellent acidic HER activity, requiring only 24 mV to reach 10 mA cm^−2^, and delivered a mass activity of 28.6 A mg^−1^ at 50 mV, over six times that of commercial Pt/C (Figure 14f). Spectroscopic and theoretical analyses uncovered that charge redistribution between Pt and O atoms within Pt‐O‐Pt units tunes the adsorption energy of H*, while the O linkers facilitate H_2_ desorption, yielding a cooperative enhancement of HER kinetics. DFT calculations further revealed that the interfacial electronic interaction induces an upshift in the Pt d‐band center relative to isolated Pt sites, strengthening the adsorption of H*. This optimized d‐band position ensures a near‐ideal Δ*G*_H*_, thereby accelerating hydrogen adsorption‐desorption kinetics. In another significant contribution, Liu et al. anchored Pt single atoms on onion‐like carbon nanospheres (Pt_1_/OLC) that exhibited excellent HER activity [142]. In‐depth analysis revealed pronounced proton accumulation around Pt centers and a strong tip‐enhanced local electric field generated by the carbon support, which collectively lowers the kinetic barrier for proton transfer during HER (Figure 14g,h). Owing to this unique microenvironment, Pt_1_/OLC (0.27 wt% Pt) exhibited outstanding acidic HER performance, characterized by a 38 mV overpotential at 10 mA cm^−2^, high mass activity, and an impressive turnover frequency of 40.78 H_2_ s^−1^ at 100 mV, surpassing graphene‐supported Pt SACs and matching the activity of Pt/C despite substantially lower Pt loading (Figure 14i).

Overall, these studies clearly demonstrate that coordination modulation through first‐shell tuning, axial ligands, and interfacial synergy directly governs HER activity by regulating key electronic descriptors such as Δ*G*_H*_, d‐band center, and charge distribution, thereby establishing a clear structure–activity relationship in SAC‐based HER catalysis.

### 6.3 Oxygen Reduction Reaction

The electrochemical ORR is a key process in energy conversion and storage technologies, including metal‐air batteries, proton‐exchange membrane fuel cells, and the electrochemical synthesis of hydrogen peroxide [165, 193, 194, 195]. ORR can proceed through two principal pathways depending on the number of electrons transferred: a two‐electron reduction route yielding H_2_O_2_, or a four‐electron pathway producing H_2_O [196]:

Two‐electron pathway with one intermediate:


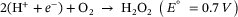

$$2\left(H^{+}+e^{-}\right)+O_{2}\;\to \;\mathrm{OO}H^{*}+\left(H^{+}+e^{-}\right)\;\to \;H_{2}O_{2}$$

Four‐electron pathway with three intermediates:


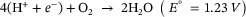

$$\begin{array}{cc} 4\left(H^{+}+e^{-}\right)+O_{2}\; & \to \;3\left(H^{+}+e^{-}\right)+\;\mathrm{OO}H^{*}\to \;2\left(H^{+}+e^{-}\right)+O^{*}\\ & \to 1\left(H^{+}+e^{-}\right)+OH^{*}\to 2H_{2}O \end{array}$$

Both pathways play important roles in practical applications; however, the sluggish reaction kinetics, large overpotentials, and limited O_2_ mass transport necessitate high Pt loadings even for state‐of‐the‐art Pt/C catalysts. The ORR activity is fundamentally governed by the adsorption energies of key intermediates (OOH*, O*, and OH*), which are closely related to electronic descriptors such as the d‐band center, charge distribution, and spin configuration of the active site. Therefore, designing catalysts that can precisely tune these descriptors to balance intermediate binding and lower the activation barrier of the rate‐determining step (RDS) remains a central challenge [197]. Tailoring the coordination environment of SACs provides an effective strategy to engineer their electronic structures and optimize oxygen intermediate binding. For instance, Zhang and coworkers demonstrated this concept by synthesizing cross‐linked polyphosphazene nanospheres with tunable hollow architectures to stabilize Co‐SACs featuring Co‐N_2_P_2_ moieties (PSTA‐Co‐1000) for alkaline ORR [198]. The PSTA‐Co‐1000 catalyst delivered a half‐wave potential of 0.88 V at 1600 rpm, outperforming Pt/C (Figure 15a). DFT calculations revealed that at a potential of 1.23 V, the Co‐N_2_P_2_ moiety significantly modifies the electronic structure of Co and possesses a lower energy barrier for the RDS (Figure 15b). Within the end‐on adsorption model, Co‐N_2_P_2_ exhibited weaker H_2_O binding but stronger O_2_ adsorption relative to Co‐N_4_, enabling more efficient O_2_ activation and facilitating the overall ORR cycle.

**FIGURE 15:**
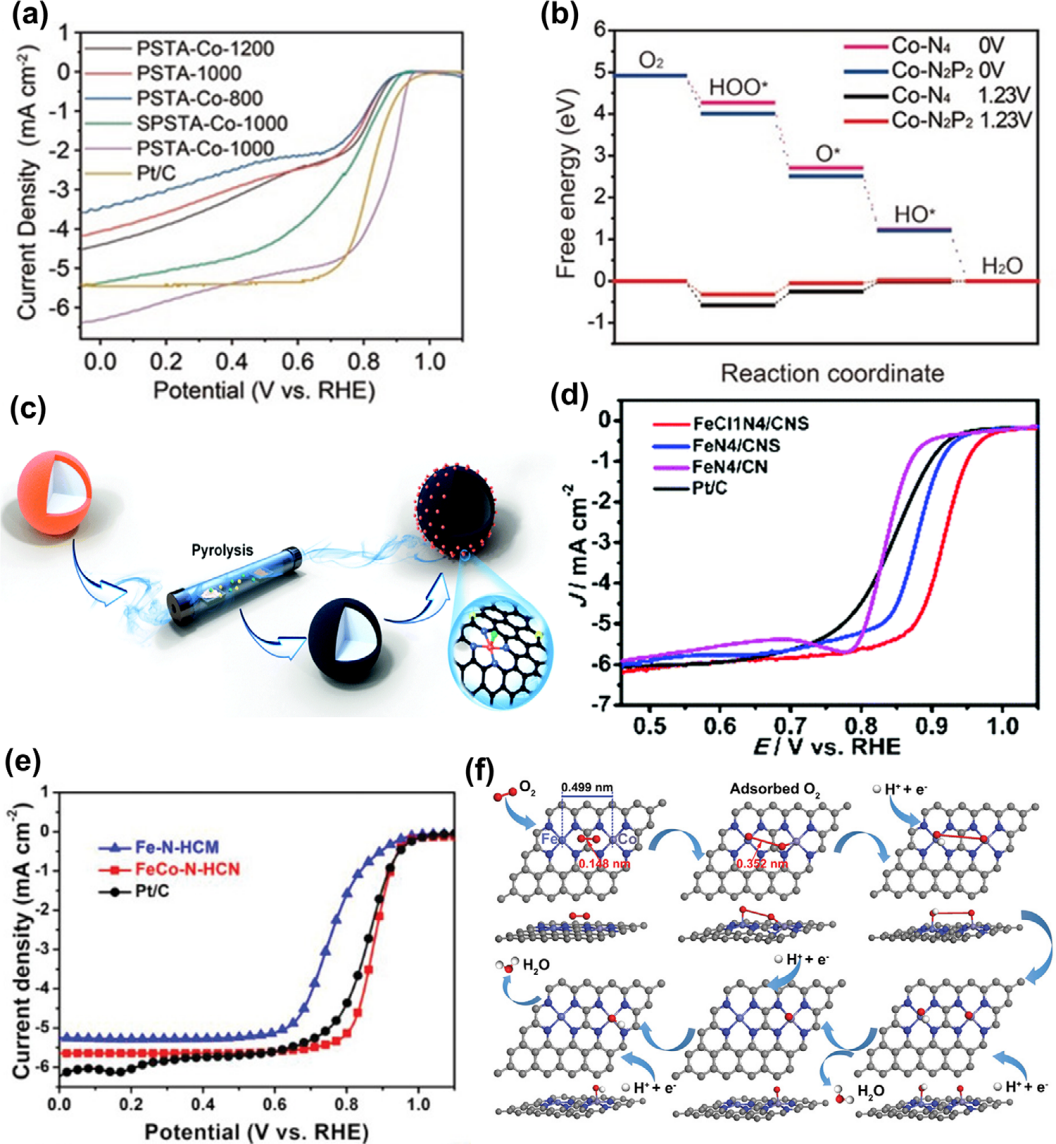
(a) The ORR LSV curves of PSTA‐Co‐T (*T*: 800°C, 1000°C, and 1200°C), PSTA‐1000, SPSTA‐Co‐1000, and Pt/C at a rotating speed of 1600 rpm in 0.1 M KOH. (b) ORR free energy diagram of Co‐N_4_ and Co‐N_2_P_2_ sites under the conditions of *U* = 0 V and *U* = 1.23 V. Reproduced with permission from ref [198]. Copyright 2020, Wiley‐VCH. (c) Schematic illustration of the synthesis of FeCl1N4/CNS. (d) ORR polarization curves in O_2_‐saturated 0.1 M KOH. Reproduced with permission from ref [199]. Copyright 2018, Royal Society of Chemistry Publication. (e) ORR LSV of the FeCo‐N‐HCN, Fe‐N‐HCM, and commercial Pt/C catalysts at 1600 rpm. (f) Proposed mechanism for ORR over the Fe‐N_4_‐C and Co‐N_4_‐C dual active centers. Reproduced with permission from ref [201]. Copyright 2021, Wiley‐VCH.

Apart from primary coordination sphere modulation, engineering the secondary coordination shell has also emerged as a powerful method to adjust local ligand fields. Qin et al. introduced S‐anion coordination into a Ru‐N‐C framework, where S atoms interact with the surrounding N ligands rather than directly coordinating with Ru [165]. This second‐shell modulation alters the electron density and ligand field around the Ru center, leading to optimized adsorption energies of oxygenated intermediates. The RuN_4_‐S units exhibit a significantly downshifted d‐band center (‐0.95 eV) compared to Ru‐N_4_ sites (‐0.15 eV), which weakens the adsorption strength of ORR intermediates and facilitates their desorption. As a result, the catalyst exhibited enhanced ORR activity and durability compared to commercial Pt/C, highlighting the importance of indirect electronic tuning. In another study, Li and coworkers subsequently demonstrated dual coordination modulation through axial Cl ligation combined with sulfur doping in the carbon substrate [199]. Using a thermal migration strategy, atomically dispersed FeCl1N4/CNS was obtained, delivering a remarkably high half‐wave potential of 0.921 V (Figure 15c,d). The synergistic interactions such as the short‐range electron withdrawal by the axial Cl ligand and long‐range electronic tuning by S, effectively reshape the Fe‐site electronic structure, optimizing intermediate adsorption and promoting faster ORR kinetics in alkaline media. Beyond isolated metal centers, adjacent dual‐metal motifs have emerged as powerful systems for further enhancing ORR activity due to cooperative electronic interactions and complementary adsorption abilities [200]. For instance, Li et al., engineered FeCo‐N‐doped hollow carbon nanocages (FeCo‐N‐HCN) featuring spatially adjacent Fe‐N_4_‐C and Co‐N_4_‐C sites [201]. Electrochemical measurements revealed outstanding ORR catalytic performance, with an onset potential of 0.98 V and a half‐wave potential of 0.86 V, surpassing Pt/C and the corresponding single‐metal SACs (Figure 15e). DFT and experimental analyses revealed that electronic coupling between Fe and Co modifies the local density of states and optimizes adsorption energies, enabling effective O‐O bond weakening while suppressing H_2_O_2_ formation. This cooperative modulation lowers the energy barriers across multiple reaction steps, thereby promoting the preferred four‐electron ORR pathway (Figure 15f).

### 6.4 Carbon Dioxide Reduction Reaction

Carbon dioxide (CO_2_), a primary greenhouse gas generated largely from fossil‐fuel combustion, is chemically inert under ambient conditions due to its highly stable linear molecular structure. Consequently, its activation and reduction require substantial energy input. Electrochemical CO_2_ reduction reaction (CO_2_RR) offers an attractive route to convert CO_2_ into value‐added chemicals and fuels by coupling renewable electricity with carbon mitigation [202, 203]. CO_2_RR performance is fundamentally governed by the adsorption energetics of key intermediates such as COOH*, OCHO*, and CO*, which are strongly dependent on electronic descriptors including d‐band center, charge distribution, and local coordination geometry. As a rapidly advancing area in energy conversion and carbon‐neutral technologies, CO_2_RR enables the formation of a range of products, primarily C_1_ species (CO, formic acid, methane) and C_2_/C_2+_ species (ethylene, ethanol, acetone), through multistep proton‐electron transfer pathways [204]. The efficiency of CO_2_RR is strongly influenced by the inherent thermodynamic stability of CO_2_ and its competition with the HER. Reaction pathways and product selectivity depend sensitively on catalyst structure, electrode potential, and the stabilization of key intermediates [205, 206]. Among these intermediates, adsorbed CO* plays a central role in both C_1_ and C_2_/C_2+_ pathways. Depending on its binding strength, CO* may desorb as CO, undergo hydrogenation to generate C_1_ products, or dimerize and further hydrogenate to form C_2_/C_2+_ species. Thus, the local coordination environment of catalytic active sites is crucial in tuning adsorption energetics and directing product distribution.

Recent research has shown that SACs exhibit remarkable advantages in CO_2_RR because of their maximized atomic utilization and highly defined active sites that provide tailored binding energies for critical intermediates [207]. In this context, the coordination modulation strategies discussed in Section 5 provide a powerful platform to engineer the local electronic structure and optimize activity, selectivity, and stability. A representative example is provided by Liu and coworkers, who introduced oxygen ligands into an Sn‐N‐C framework to generate a series of Sn SACs with precisely controlled coordination environments for promoting CO_2_‐to‐HCOOH conversion (Figure 16a) [208]. The room‐temperature Sn Mössbauer spectroscopy revealed that O incorporation oxidized Sn(II) entirely to Sn(IV), forming O‐Sn‐N_4_ moieties. These axially oxygen‐coordinated Sn(IV) sites exhibited exceptional selectivity toward formate, achieving a high Faradaic efficiency of 89.4% at ‐1.0 V versus RHE (Figure 16b). From a descriptor perspective, the introduction of axial O ligands induces asymmetric charge redistribution and effectively modifies the spin configuration, optimizing the adsorption configuration of CO_2_* and stabilizing the OCHO* intermediate. Operando XAFS and additional in situ characterizations identified the formation of surface‐bound bidentate tin carbonate species (Sn‐O‐CO_2_) during CO_2_RR, demonstrating dynamic restructuring of the coordination and electronic environment at the active sites (Figure 16c,d). DFT calculations confirmed that this coordination environment lowers the energy barrier for OCHO* formation and subsequent hydrogenation, thereby accelerating CO_2_ conversion to HCOOH. In another study, Chen and coworkers demonstrated that Fe‐based SACs can significantly enhance CO_2_RR performance through second‐shell coordination engineering [209]. They synthesized an Fe_1_‐NSC catalyst featuring Fe‐N_4_S moieties, in which sulfur atoms occupy the second coordination shell of the isolated Fe centers (Figure 16e). This modification not only tunes the electronic structure of Fe centers but also induces an upshift of the d‐band center toward the Fermi level, strengthening the interaction with the COOH* intermediate and facilitating CO_2_ activation. Consequently, the regulated electronic structure promotes favorable CO_2_ adsorption and lowers the energy barrier for the formation of COOH*. Additionally, the presence of S lowers the dissociation free energy of H_2_O at adjacent carbon sites, enabling an efficient proton‐feeding effect that accelerates subsequent protonation steps. As a result, the Fe_1_‐NSC catalyst exhibited superior CO_2_RR performance relative to sulfur‐free Fe‐N_4_ analogues and most previously reported Fe‐based SACs, delivering a maximum CO Faradaic efficiency of 98.6% (Figure 16f).

**FIGURE 16:**
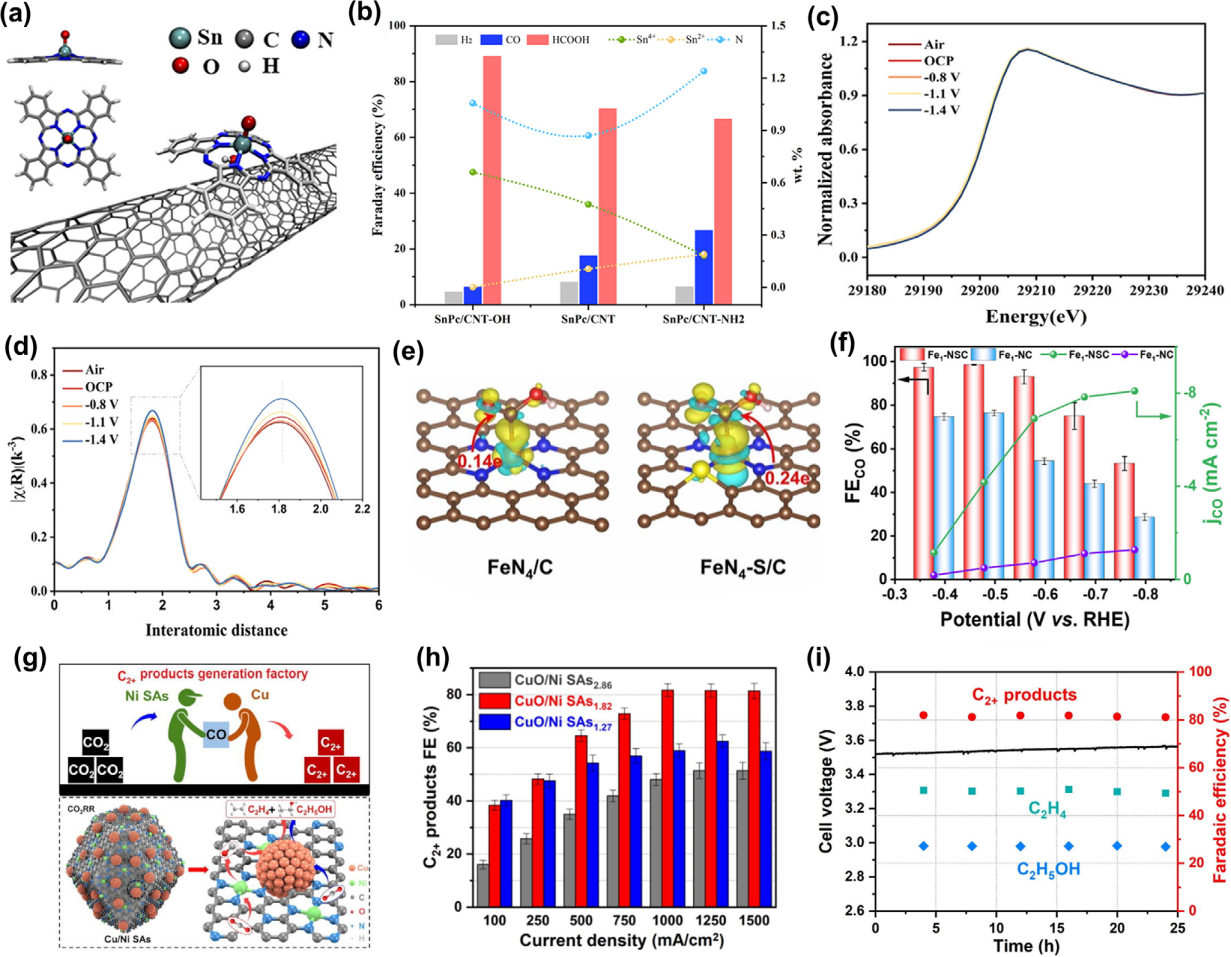
(a) Schematic illustration for the preparation of SnPc/CNT‐OH. (b) Correlation diagram of the CO_2_RR Faradaic efficiency and the relative contents of Sn(II), Sn(IV), and nitrogen species of SnPc/CNT, SnPc/CNT‐OH, and SnPc/CNT‐NH_2_: FE is indicated as columns, while Sn(II), Sn(IV), and N content are indicated as curves. (c) Normalized operando Sn K‐edge XANES spectra. (d) FT magnitude of EXAFS spectra of SnPc/CNT‐OH during CO_2_RR recorded in the CO_2_‐saturated 0.5 M KHCO_3_ solution. Reproduced with permission from ref [208]. Copyright 2023, American Chemical Society. (e) Differential charge density distribution between the catalyst (FeN_4_/C and FeN_4_‐S/C) and *COOH intermediates. (f) FE_CO_ and j_CO_ of Fe_1_‐NSC and Fe_1_‐NC at different potentials in CO_2_‐saturated electrolyte. Reproduced with permission from ref [209]. Copyright 2022, Wiley‐VCH. (g) Schematics of the CuO/Ni SAs tandem catalyst and functioning mechanisms. (h) The C_2+_ products comparisons for tandem catalysts of CuO/Ni SAs_2.86_, CuO/Ni SAs_1.82_, and CuO/Ni SAs_1.27_. (i) The CO_2_RR stability of the MEA cell equipped with CuO/Ni SAs tandem catalyst. Reproduced with permission from ref [210]. Copyright 2022, Elsevier.

Beyond single‐site optimization, tandem catalytic strategies closely related to interfacial synergy concepts discussed in Section 5.5 have emerged as an effective route for promoting multi‐carbon (C_2+_) product formation by decoupling the overall CO_2_RR into sequential CO_2_‐to‐CO and CO‐to‐C_2+_ steps. Wu and coworkers designed a CuO/Ni single‐atom tandem electrocatalyst in which adjacent Ni single atoms and CuO domains independently catalyze CO generation and C‐C coupling, respectively (Figure 16g) [210]. This spatially proximate configuration enables the in‐situ generation and rapid consumption of CO intermediates. From an electronic perspective, the distinct coordination environments of Ni and Cu sites provide complementary adsorption strengths for CO* and C–C coupling intermediates, effectively overcoming scaling limitations. As a result, the CuO/Ni single‐atom tandem catalyst achieved an exceptionally high partial current density for C_2+_ products of 1220.8 mA cm^−2^, while maintaining a high C_2+_ Faradaic efficiency of 81.4% and excellent selectivity toward ethylene (54.1%) and ethanol (28.8%) (Figure 16h). The superior performance was attributed to the minimized distance between Ni single atoms and CuO active sites, which enhances CO utilization efficiency and suppresses mass‐transport limitations. Notably, in a membrane electrode assembly (MEA)‐based CO_2_ electrolyzer, the CuO/Ni tandem catalyst delivered a C_2+_ Faradaic efficiency of 82.1% with stable operation over 25 h (Figure 16i), providing valuable design principles for next‐generation tandem catalysts aimed at scalable C_2+_ production from CO_2_RR.

Overall, these studies clearly establish that coordination environment modulation through axial ligands, second‐shell tuning, and interfacial/tandem design directly governs CO_2_RR performance by regulating key electronic descriptors such as d‐band center, charge distribution, and intermediate adsorption energies. This provides a mechanistic framework linking coordination structure to activity and selectivity, addressing the fundamental structure–activity relationships emphasized throughout this review.

## 7 Summary and Perspectives

SACs represent an emerging class of catalytic materials characterized by maximized metal utilization and well‐defined geometric and electronic structures. By integrating the advantages of homogeneous catalysis, such as uniform active sites and tunable electronic properties, with the robustness and recyclability of heterogeneous systems, SACs have demonstrated exceptional potential for energy‐related catalytic applications. As emphasized throughout this review article, the catalytic behavior of SACs is fundamentally governed by their local coordination environments, which dictate key electronic descriptors such as the d‐band center, charge distribution, and spin configuration of the isolated metal centers. These descriptors, in turn, regulate the adsorption energetics of reaction intermediates and determine activity, selectivity, and stability across important electrocatalytic processes including OER, HER, ORR, and CO_2_RR.

A central theme of this review is that coordination environment engineering provides a unifying framework to rationally design SACs by linking atomic structure to catalytic function through electronic descriptors. In contrast to conventional reviews that primarily focus on synthetic methodologies or performance comparisons, we highlight here how first and second‐shell coordination, MSIs, and interfacial effects collectively tune the electronic structure of active sites and thereby control reaction pathways. From the studies discussed, several general design principles can be identified: (i) Lowering the coordination number or introducing unsaturated sites typically upshifts the d‐band center, strengthening intermediate adsorption, but requires careful balance to avoid too strong binding. (ii) Second‐shell or heteroatom modulation enables subtle electronic tuning via modulating the local ligand field without compromising the structural stability. (iii) Axial ligands and asymmetric coordination environments can break conventional scaling relationships via precisely modulating the spin‐states. (iv) Dual‐atom or interfacial motifs introduce cooperative effects that optimize multi‐step reaction pathways.

Despite the significant progress achieved in regulating the coordination environments of SACs, several critical challenges remain unresolved. Addressing these issues is essential for advancing fundamental understanding and enabling the translation of SACs from laboratory‐scale studies to practical energy conversion and storage technologies: (i) Under realistic electrochemical operating conditions, SACs often undergo dynamic structural evolution, including changes in coordination number, bond distances, and oxidation/spin states. As a result, the true active sites may differ from those identified under ex situ conditions. Advanced operando spectroscopic and microscopic techniques with high spatial and temporal resolution are, therefore, essential to capture these dynamic transformations and establish reliable structure–activity relationships. (ii) The intrinsic heterogeneity of practical supports leads to a distribution of coordination environments, making it difficult to precisely correlate structure with performance. Developing synthetic strategies that yield well‐defined and uniform coordination motifs remains a key challenge. (iii) The typically low metal loadings in SACs present a major limitation for achieving high reaction rates, thereby restricting their practical applicability. To satisfy the demands of both fundamental research and industrial implementation, it is imperative to increase single‐atom loadings while maintaining uniform dispersion, strong anchoring, and structural stability. The realization of scalable, green, and high‐loading SAC synthesis routes represents a key direction for future development and performance enhancement. (iv) The long‐term durability of many SACs remains inadequate, as isolated metal atoms possess high surface free energies and can readily undergo migration, aggregation, or undesired side reactions under operating conditions. Developing robust stabilization strategies that suppress atom migration while preserving catalytic activity is therefore essential for improving the operational lifetime of SACs.

In addition, owing to their atomically uniform active sites, SACs also hold promise as emerging spin‐dependent catalysts. Spin polarization at single‐atom sites can influence adsorption energetics and reaction pathways, particularly in reactions involving spin‐sensitive intermediates, yet its role remains insufficiently understood under operando conditions. Similarly, the interplay between the coordination environment and the electrolyte/electric double layer is often overlooked, despite its significant influence on catalytic behavior.

With the rapid expansion of SAC research, several critical pitfalls in evaluating electrocatalytic performance have emerged. In particular, the identification and quantification of true active sites remain challenging, and normalization of catalytic activity (e.g., turnover frequency) is often based on assumptions that may not accurately reflect the number of accessible active centers. In addition, contributions from support materials, hidden aggregations, trace metal impurities, or dynamically formed species under reaction conditions can lead to misleading interpretations of intrinsic activity. Establishing standardized protocols combining rigorous site quantification, operando characterization, and consistent benchmarking metrics will be essential for ensuring reliable and reproducible evaluation of SAC performance.

Looking forward, the integration of advanced characterization, theory, and data‐driven approaches is expected to accelerate the rational design of SACs. In particular, artificial intelligence and machine learning offer powerful tools to establish quantitative relationships between coordination environments, electronic descriptors, and catalytic performance. By combining high‐throughput computations with experimental validation, these approaches can enable rapid screening of optimal coordination motifs, prediction of stability under realistic conditions, and discovery of new catalyst systems. Furthermore, the development of scalable and controllable synthesis methods, coupled with operando insights, will be essential for translating SACs from model systems to practical devices.

In summary, the deliberate modulation of local coordination environments provides a powerful strategy to control the electronic structure and catalytic behavior of SACs at the atomic level. By establishing clear links between coordination structure, electronic descriptors, and catalytic performance, this review outlines a mechanistic framework for the rational design of next‐generation electrocatalysts. Continued efforts in precise synthesis, advanced characterization, and theory‐guided design are expected to unlock the full potential of SACs for sustainable energy conversion and chemical transformations.

## Author Contributions

**Ashwani Kumar**: conceptualization, writing – original draft. **Harun Tüysüz**: conceptualization, resources, writing – review and editing, supervision.

## Conflicts of Interest

The authors declare no conflicts of interest.

## Data Availability

Data sharing not applicable to this article as no datasets were generated or analysed during the current study.
